# Ginsenoside Rg3 enriches SCFA-producing commensal bacteria to confer protection against enteric viral infection via the cGAS-STING-type I IFN axis

**DOI:** 10.1038/s41396-023-01541-7

**Published:** 2023-11-10

**Authors:** Gan Wang, Jingtianyi Liu, Yanan Zhang, Jinyan Xie, Shuxian Chen, Yuhua Shi, Fushan Shi, Shu Jeffrey Zhu

**Affiliations:** Department of Veterinary Medicine, College of Animal Sciences, Zhejiang University, Hangzhou, 310058 Zhejiang PR China

**Keywords:** Virus-host interactions, Microbiome

## Abstract

The microbiota-associated factors that influence host susceptibility and immunity to enteric viral infections remain poorly defined. We identified that the herbal monomer ginsenoside Rg3 (Rg3) can shape the gut microbiota composition, enriching robust short-chain fatty acid (SCFA)-producing *Blautia* spp. Colonization by representative *Blautia coccoides* and *Blautia obeum* could protect germ-free or vancomycin (Van)-treated mice from enteric virus infection, inducing type I interferon (IFN-I) responses in macrophages via the MAVS-IRF3-IFNAR signaling pathway. Application of exogenous SCFAs (acetate/propionate) reproduced the protective effect of Rg3 and *Blautia* spp. in Van-treated mice, enhancing intracellular Ca^2+^- and MAVS-dependent mtDNA release and activating the cGAS-STING-IFN-I axis by stimulating GPR43 signaling in macrophages. Our findings demonstrate that macrophage sensing of metabolites from specific commensal bacteria can prime the IFN-I signaling that is required for antiviral functions.

## Introduction

Cumulative evidence supports that the intestinal microbiome can inhibit viral infections both locally and systemically through the release of microbial metabolites [1–4]. For example, Winkler et al. demonstrated that *Clostridium scindens* can modulate prompt type I interferon (IFN-I) responses to prevent Chikungunya virus from infecting and disseminating in host blood monocytes via primary to secondary bile acid transformation [4]. In addition, one *Clostridium orbiscindens*-derived flavonoid degradation product, desaminotyrosine, has been shown to augment IFN-I responses in pulmonary macrophages and protect mice from influenza A virus (IAV) infection [2].

Short-chain fatty acids (SCFAs) including acetate, propionate and butyrate are mainly generated in the colon by bacterial fermentation of undigested complex carbohydrates [5]. SCFAs are generally considered to elicit anti-inflammatory properties either by binding to the G protein-coupled receptors GPR41 (also known as FFAR3) and GPR43 (also known as FFAR2), or by inhibiting histone deacetylases in different cell types [6, 7]. SCFAs can impact viral infections both in vitro [8] and in vivo [9]. For instance, acetate or dietary fiber-derived butyrate can exert protective effects against IAV infection by inducing IFN-I responses [10] or reducing neutrophil infiltration into the airways [11].

Although the gut microbiome has been described to play a role in regulation of innate and adaptive antiviral immune responses [12–14], very little is known about the interplay between SCFA-producing commensal bacteria, the host immune system and infecting enteric viruses. In a previous study we demonstrated that the intestinal microbiome primes IFN-I induction in mononuclear phagocytes to antagonize systemic encephalomyocarditis virus (EMCV) infection via an IFNAR- and STAT1-mediated signaling pathway [15]. Although the acetogenic bacterium *Blautia coccoides* has been identified to confer protection against enteric viruses in an IFN-I-dependent manner, whether SCFAs can regulate IFN-I signaling in mononuclear phagocytes remains unknown. Furthermore, a mechanistic understanding of the signaling pathways linking host antiviral IFN-I immune responses and microbial-derived SCFAs during enteric virus infection is still lacking.

Ginsenosides are the main active components of ginseng, an ancient herb that has been used extensively worldwide as a natural tonic [16]. Ginsenosides Rb1 and Rg1 have previously been shown to restrict murine norovirus (MNV) replication in vitro [17]. Here, we describe notable enrichment of SCFA-producing commensal bacteria *Blautia* spp. in mice following ginsenoside Rg3 (Rg3) consumption. Acetate and propionate, which are known metabolites of *Blautia* spp. primed IFN-I-mediated innate antiviral immunity to enteric viral challenge at mucosal and extraintestinal sites via GPR43-cGAS-STING signaling.

## Results

### Rg3 elicits protection against local and systemic enteric virus infection by regulating the intestinal microbiome

**Fig. 1:**
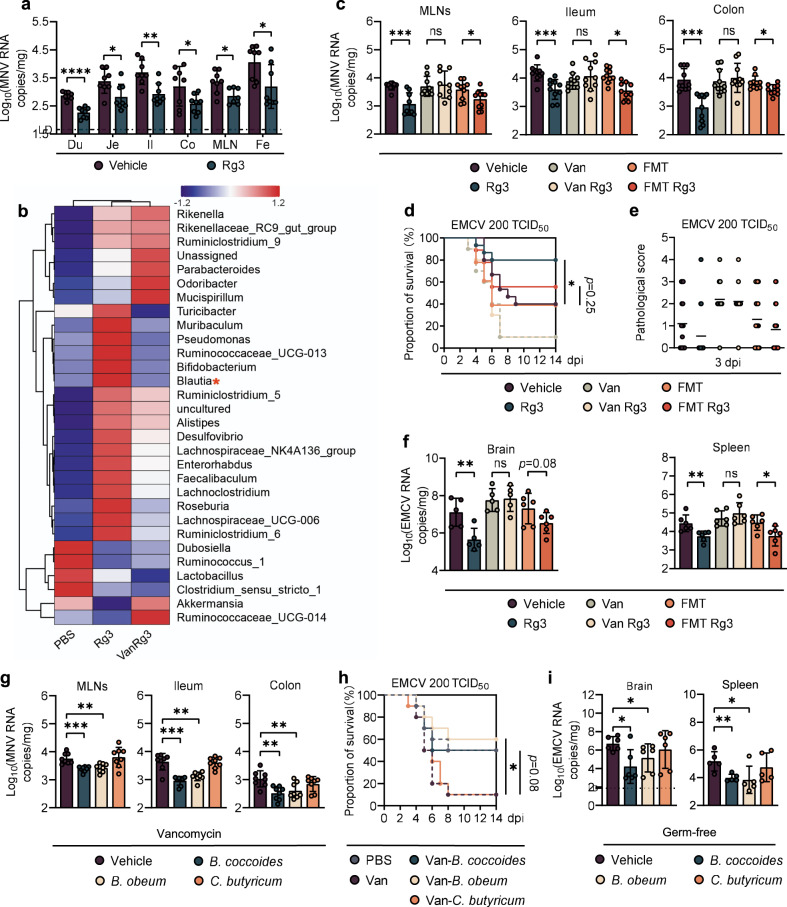
Rg3 elicits protection against local and systemic infection of enteric viruses by enriching commensal bacteria. **a** Viral burden analysis at 1 day post-infection (dpi) of duodenum (Du), jejunum (Je), ileum (Il), colon (Co), mesenteric lymph nodes (MLNs) and feces (Fe) collected from vehicle-treated or Rg3-treated mice infected with 2 × 10^7^ TCID_50_ MNV (*n* = 8). **b** Heatmap of the relative abundance of bacteria in feces from MNV-infected mice at 0 dpi (n > 3). **c** Viral burden analysis of MLNs, ileum and colon tissues collected at 1 dpi from vehicle-, Rg3-, Van-, Van-Rg3-, FMT- treated or FMT-Rg3-treated mice that were orally inoculated with 2 × 10^7^ TCID_50_ MNV (*n* = 8). Survival curve (**d**) and pathology scores (**e**) from groups of differently treated mice i.p. infected with 2 × 10^2^ TCID_50_ of EMCV (*n* = 15–18). **f** Viral titers in brain (left) and spleen (right) collected from differently treated mice at 3 dpi following i.p. infection with 1 × 10^3^ TCID_50_ of EMCV (*n* = 6). **g** Viral burden at 1 dpi in MLNs, ileum and colon tissues collected from Van-treated mice that were subsequently colonized with *B. coccoides, B. obeum*, *C. butyricum* and then inoculated orally with 2 × 10^7^ TCID_50_ MNV (*n* = 8). **h** Survival curves from groups of PBS-, Van-treated and bacteria-colonized WT mice that were i.p. infected with 2 × 10^2^ TCID_50_ of EMCV (*n* = 10). **i** Viral burden at 3 dpi in brain and spleen of germ-free (GF) mice colonized with *B. coccoides, B. obeum* or *C. butyricum* for 48 h and then i.p. infected with 2 × 10^2^ TCID_50_ EMCV (*n* = 5–6). Broken lines indicate the limit of detection (LD). Plotted data represent the mean ± s.d. Unpaired two-tailed Student’s *t* tests were used for comparison of means between treatment groups. *p <0.05; **p < 0.01; ***p < 0.001; ****p < 0.0001; ns not significant.

To assess the antiviral effects of Rg3 in vivo, groups of C57BL/6J mice (referred to as WT B6 hereafter) were orally administered Rg3 for 3 consecutive days, inoculated with MNV-1 by the peroral route, and viral burden in intestinal tissues was determined at 1 day post-infection (dpi). Rg3 treatment led to diminished viral replication in the duodenum, jejunum, ileum, colon, mesenteric lymph nodes (MLNs) and also resulted in decreased fecal viral shedding relative to the untreated controls (Fig. 1a). Additionally, Rg3 treatment of mice for 7 or 14 days prior to infection resulted in significantly lower viral titers in the intestinal tissues, MLNs and feces compared with untreated controls, in a time-course dependent manner (Supplementary Fig. S1A). This phenotype was not due to a direct virostatic effect of Rg3, since Rg3-pretreated primary bone marrow-derived macrophages (BMDMs) showed no reduction of viral titers in vitro after MNV-1 infection at different multiplicities of infection (MOI) (Supplementary Fig. S1B).

To test if the protective antiviral effects exerted by Rg3 in vivo were due to alterations of the intestinal microbiome, we first performed 16S rRNA sequencing on stools from PBS- or Rg3-treated mice. Global community composition at the family level indicated that Rg3 administration induced a considerable increase in the proportion of *Lachnospiraceae* and *Bifidobacteriaceae* together with a significant reduction in the numbers of operational taxonomic units (OTUs) belonging to the Akkermansiaceae and Lactobacillaceae families (Supplementary Fig. S1C). In particular, well-known SCFA-producers including *Blautia*, *Faecalibaculum* and *Lachnoclostridium* were the main enriched genera in the Rg3-treated mice compared with their PBS-treated counterparts (Fig. 1b).

We performed microbiota depletion studies to further confirm the hypothesis that Rg3 alterations to the intestinal flora confers protection against enteric virus infection. Groups of WT B6 mice were gavaged with vancomycin (Van), neomycin (Neo), ampicillin (Amp), or metronidazole (Metro) for 5 days and subsequently treated with Rg3 for 3 days before MNV-1 inoculation. Depletion of the gut microbiota by antibiotic treatment was observed as a significant reduction in 16S rRNA gene copies (Supplementary Fig. S1D), and a greatly altered enteric bacterial community composition before infection (Supplementary Fig. S1E). Van was selected for subsequent studies due to its high efficiency in depleting Gram-positive, anaerobic, fermentative, SCFA-producing spp. of the *Lachnospiraceae* family (Fig. 1b).

The MNV-1 inhibitory effect of Rg3 treatment was abolished when mice were pretreated with Van, whereas reconstitution of the gut flora by fecal microbiota transplantation (FMT) from antibiotic-naive, uninfected mice resulted in decreased MNV-1 titers in the ileum, colon and MLNs of the Rg3-treated mice (Fig. 1c). As expected, FMT restored the 16S rRNA gene copy numbers that had been substantially reduced by Van treatment (Supplementary Fig. S1F).

We tested the protective effect of Rg3 treatment against EMCV challenge, a more severe viral infection model that results in severe encephalitis. Similar to MNV-1, Rg3 pretreatment did not impair EMCV replication in RAW cells (Supplementary Fig. S1G). However, a 3-day oral Rg3 administration prior to intraperitoneal (i.p.) EMCV injection led to a marked decrease in lethality (Fig. 1d) and alleviated neurovirulence compared with nontreated controls (Fig. 1e). This protective effect was abolished in Van-treated mice, and partially restored by FMT; there was a trend in lower lethality and neurovirulence for FMT plus Rg3 treatment versus FMT alone, although it was not statistically significant (Fig. 1d, e). In support of this, we observed that Van treatment vanished the inhibitory effect exerted by Rg3 on viral replication in the spleen and brain of EMCV-infected PBS mice, while microbiome reconstitution by FMT in Van-treated mice protected them from EMCV replication in these tissues in the presence of Rg3 (Fig. 1f). In summary, these data suggest that Rg3 can limit local and systemic enteric virus infections and ameliorate the resulting disease in a gut microbiome-dependent manner.

### The protective effect of Rg3 against enteric virus infection results from enrichment of commensal *Blautia* spp.

Since Rg3 administration stimulated the growth of certain bacterial communities from the *Blautia* genus (Fig. 1b), we chose *B. coccoides* and *B. obeum* as representative isolates to determine how they protect against enteric virus infection. Van-treated mice were colonized with *B. coccoides* or *B. obeum* and then infected with MNV-1. *Clostridium butyricum*, which is a robust butyrate-producer that confers no protection against EMCV systemic infection [15], served as an unrelated Van-sensitive, Gram-positive control. As expected, colonization of Van-treated mice with *B. coccoides* or *B. obeum* markedly reduced the MNV-1 viral burden in the ileum, colon and MLNs whereas *C. butyricum* gavage failed to do so despite effective colonization (Fig. 1g and Supplementary Fig. S2A).

For EMCV protection assays, Van-treated mice were gavaged with *B. coccoides*, *B. obeum* or *C. butyricum* prior to i.p. EMCV inoculation. Survival rates increased from 10% in Van-treated mice to 50% and 60% in Van-treated animals colonized by *B. coccoides* or *B. obeum*, respectively, which was similar to the 40% survival rate of PBS control mice. *C. butyricum* did not alter EMCV lethality in Van-treated mice, despite effective colonization (Fig. 1h and Supplementary Fig. S2A). Moreover, Van-treated mice colonized with *B. coccoides* or *B. obeum* but not *C. butyricum* exhibited remarkably reduced viral titers in the brain and spleen compared with noncolonized controls (Supplementary Fig. S1I).

To further confirm a specific role for the commensal *B. coccoides* and *B. obeum* in modulating enteric virus infections, we repeated the colonization and EMCV infection studies in a germ-free (GF) mouse model. Following efficient colonization of GF mice (Supplementary Fig. S2B), significantly lower EMCV titers were detected at 3 dpi in the brain and spleen of mice colonized with *B. coccoides* or *B. obeum* but not *C. butyricum* (Fig. 1i). These concordant results in Van-treated and GF mice indicate that *Blautia* spp. can specifically modulate enteric virus infection both at mucosal and systemic sites.

### *Blautia*-associated protection against enteric virus infection results from increased acetate and propionate concentrations

**Fig. 2:**
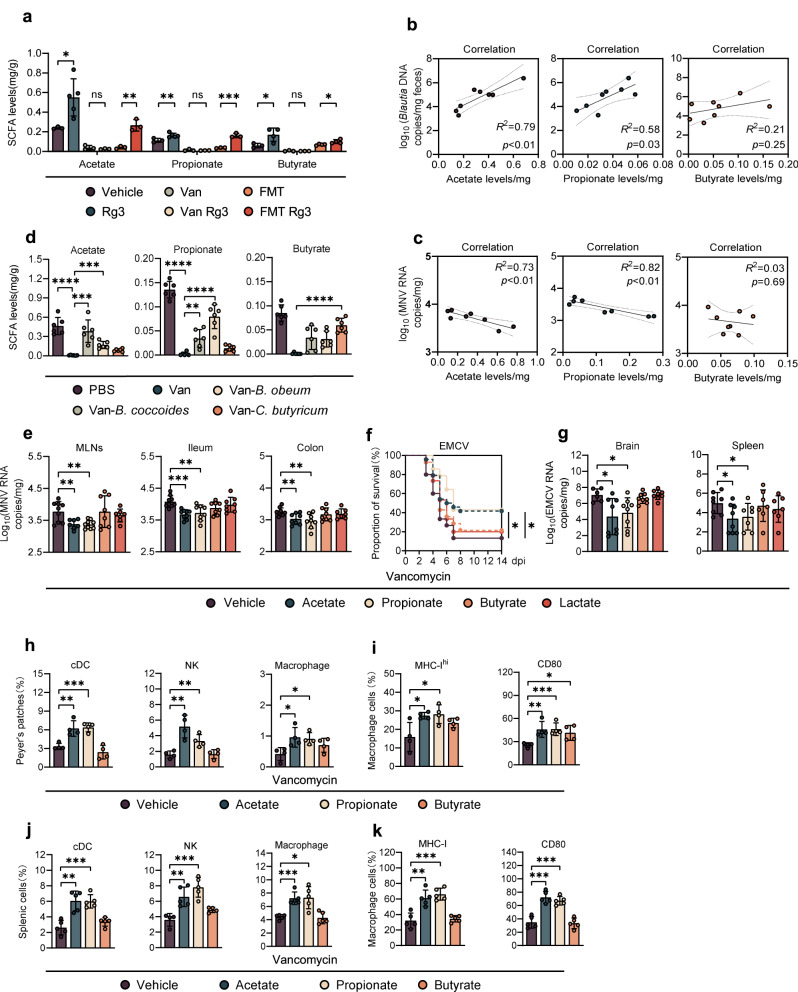
Rg3 restricts enteric virus infection by enhancing *Blautia*-associated acetate and propionate. **a** Fecal SCFA concentration of vehicle-, Rg3-, Van-, Van-Rg3-, FMT- treated or FMT-Rg3-treated, uninfected mice (*n* = 3–4). **b** Correlation between relative fecal abundance of *Blautia* spp. and acetate (left), propionate (mid), or butyrate (right) concentration in Van-treated, *Blautia*-colonized mice (*n* = 8). **c** Correlation between fecal acetate (left), propionate (mid), butyrate (right) concentration and MNV viral burden in the ileum of Van-treated, *Blautia-*colonized mice (*n* = 8). **d** Fecal acetate (left), propionate (mid) and butyrate (right) concentration of Van-treated and *Blautia*- or *C. butyricum*-colonized, uninfected mice (*n* = 5–6). **e** Viral burden analysis at 1 dpi of MLNs, ileum and colon tissues collected from mice that were pretreated for 2 weeks with 200 mM acetate, propionate or butyrate in drinking water prior to oral infection with 2 × 10^7^ TCID_50_ MNV (*n* = 8). **f** Survival curves of groups of Van-treated mice pretreated with 200 mM acetate, propionate or butyrate prior to i.p. infection with 2 × 10^2^ TCID_50_ of EMCV (*n* = 13–24). **g** Viral titers at 3 dpi in brain and spleen collected from Van-treated mice pretreated with 200 mM acetate, propionate or butyrate prior to i.p. infection with 1 × 10^3^ TCID_50_ of EMCV (*n* = 6–8). **h**–**k** Frequency of a panel of innate immune cells including conventional dendritic cells (cDCs), NK cells and macrophages from (**h**) the distal ileum PPs of MNV-1-infected mice at 1 dpi or (**j**) spleen of EMCV-infected mice at 3 dpi. Expression of MHC-I and CD80 on (**i**) PP macrophages from differently treated and MNV-infected mice at 1 dpi, or (**k**) splenic macrophages from differently treated and EMCV-infected mice at 3 dpi (*n* = 5–6). Plotted data represent the mean ± s.d. *R*^*2*^ and exact two-sided *p* values calculated by Pearson’s test are shown. Unpaired two-tailed Student’s *t* tests were used for statistical purposes. *p < 0.05; **p < 0.01; ***p < 0.001, ****p < 0.0001.

Given the significant increase in relative abundance of SCFA-producing bacteria in the mice administered Rg3 (Fig. 1b), we wanted to measure the changes in actual SCFA production. Using GC-MS to detect absolute SCFA concentrations in mouse fecal samples, we found a significant enhancement of SCFA after 3 days of oral Rg3 administration. This enhancement was abolished by Van treatment and partially reconstituted by FMT (Fig. 2a). Concentrations of acetate and propionate, but not butyrate, positively correlated with *Blautia* abundance (Fig. 2b), and negatively correlated with intestinal MNV-1 titers or splenic EMCV viral burden (Fig. 2c and Supplementary Fig. S3B).

To further confirm the relationship between *Blautia* spp. and increased acetate/propionate production, we colonized Van-treated mice with *B. coccoides*, *B. obeum* or *C. butyricum* and quantified fecal SCFAs at 48 h post-colonization. *B. coccoides*, *B. obeum* and *C. butyricum* mostly restored the fecal concentration of acetate, propionate or butyrate, respectively, and partially rescued production of other SCFAs (Fig. 2d and Supplementary Fig. S3A). Consistently, we analyzed bacterial cultures of *B. coccoides*, *B. obeum* and *C. butyricum* with GC-MS and discovered a similar SCFA production to that observed in the in vivo colonization assay (Supplementary Fig. S3C).

To corroborate the protective role of SCFAs against local enteric virus infection, we directly administered acetate, propionate or butyrate in the drinking water of Van-treated mice for 2 weeks prior to enteric virus infection. Lactate was used as an unrelated glucose metabolite control. Fecal SCFA concentrations were significantly higher in the treated groups compared with the nontreated controls (Supplementary Fig. S3D); a significant reduction in intestinal and lymphatic MNV-1 burden was observed in mice given acetate or propionate, but not butyrate or lactate (Fig. 2e). In the more severe systemic EMCV infection model, acetate/propionate pretreatment reduced mortality by more than 20% (Fig. 2f), viral titers in the brain and spleen were remarkably decreased (Fig. 2g), and neuropathology was significantly ameliorated (Supplementary Fig. S3E). In contrast, butyrate or lactate treatment did not protect against EMCV based on survival curves, tissue viral burden or cerebral lesions (Fig. 2e, f, Supplementary Fig. S3E). Wild-type (WT) control mice treated with acetate or propionate had lower EMCV-induced lethality, and butyrate treatment even exacerbated mortality (Supplementary Fig. S3F).

### SCFA-related protection from enteric virus infection is dependent on macrophages

To identify the cell types that participate in the early control of MNV-1 infection mediated by SCFAs, we first evaluated the recruitment and activation of early responding innate immune cells in the distal ileum Peyer’s patches (PPs) at 1 dpi of MNV-1 infection by flow cytometry (Supplementary Fig. S4A). We observed a significant increase in the percentage of NK cells, macrophages, and conventional dendritic cells (cDCs) in Van-treated mice given acetate or propionate, but not butyrate compared with the PBS-treated control mice (Fig. 2h). In contrast, neither Van nor SCFA treatment altered the frequencies of T-cells or B-cells in the context of MNV-1 infection (Supplementary Fig. S4C). Furthermore, we discovered that pretreatment with acetate or propionate but not butyrate significantly augmented surface molecules associated with macrophage activation, such as MHC-I and CD80, upon MNV-1 infection (Fig. 2i).

Three days after EMCV infection, proportions of NK cells, macrophages and cDCs were greatly increased in the spleen of Van-treated mice given acetate or propionate but not butyrate prior to infection, compared with the PBS-treated controls (Fig. 2j and Supplementary Fig. S4B). Similar to MNV-1 infection, frequencies of T/B lymphocytes were not altered in the SCFA-treated mice (Supplementary Fig. S4D). MHC-I and CD80 were also upregulated 3 days after EMCV infection in the splenic macrophages from Van-treated mice given acetate or propionate but not butyrate (Fig. 2k).

To further determine the role of mononuclear phagocytes in SCFA-related protection against acute enteric virus infection, we depleted mononuclear phagocytes in Van-treated mice using clodronate liposomes and analyzed survival after EMCV inoculation. Clodronate greatly impaired the protection mediated by acetate and propionate treatment, suggesting that acetate/propionate-driven antiviral effect might be correlated to the activation of mononuclear phagocytes (Fig. 2f and Supplementary Fig. S4E). Furthermore, the protective patterns of acetate and propionate treatment were comparable between Van-treated WT B6 and *Rag1*^*−/−*^ mice (lacking all T and B cell responses), suggesting that lymphocytes and adaptive immunity are dispensable for SCFA-driven protection against EMCV systemic infection (Supplementary Fig. S4F).

### Rg3- and *Blautia*-derived acetate and propionate restrict enteric virus infection by stimulating IFN-I responses in macrophages

**Fig. 3:**
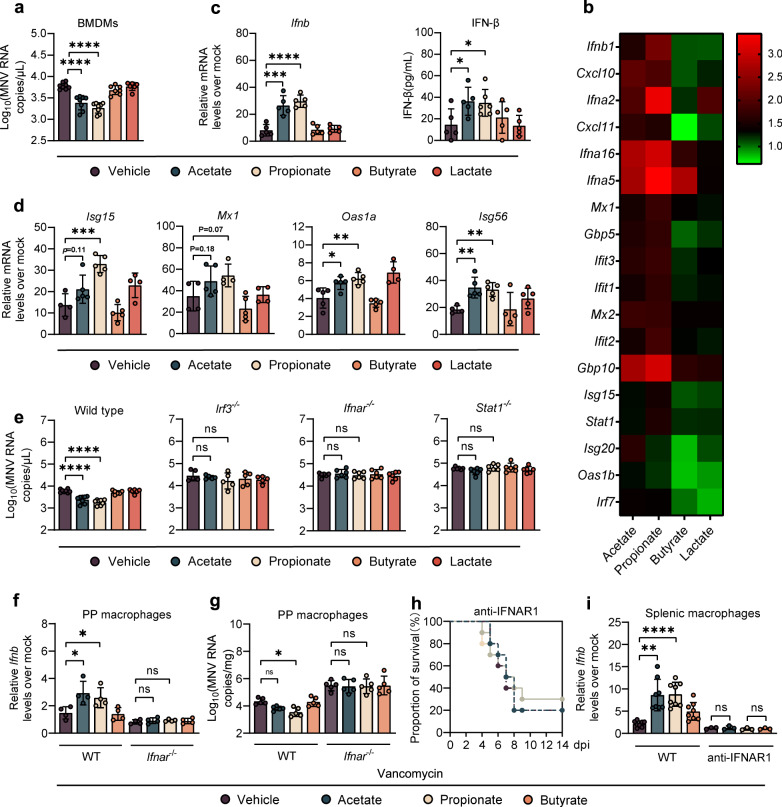
Acetate and propionate restrict enteric virus infection by stimulating IFN-I responses in macrophages. **a** Viral titers at 1 dpi in BMDMs from WT mice pretreated with 1 mM acetate, propionate, butyrate or 10 mM lactate for 24 h prior to MNV infection (MOI = 1) (*n* = 6–8). **b** Heatmap profiling the expression of 18 genes involved in IFN-β and ISGs. **c** Relative *Ifnb* expression (left, fold change compared with mock-infected BMDMs) and IFN-β protein (right) determined at 8 hpi in SCFA-treated BMDMs after MNV infection (*n* = 5–6). **d** Relative expression of different ISGs at 8 hpi in BMDMs pretreated for 24 h with 1 mM acetate, propionate, butyrate, or 10 mM lactate prior to MNV infection (*n* = 4–5). **e** Viral titers at 1 dpi in BMDMs collected from WT, *Irf3*^−/−^, *Ifnar*^−/−^, or *Stat1*^−/−^ mice pretreated with 1 mM acetate, propionate, butyrate, or 10 mM lactate prior to MNV infection (*n* = 5–6). Relative *Ifnb* expression (**f**) and viral burden (**g**) at 1 dpi in PP macrophages sorted from Van- and SCFA-pretreated, MNV-infected WT or *Ifnar*^−/−^ mice (*n* = 4–5). **h** Survival curves for groups of Van- and SCFA-pretreated mice treated with anti-IFNAR1 IgG prior to i.p. infection with EMCV (*n* = 8). **i** Relative *Ifnb* expression in splenic macrophages at 3 dpi sorted from Van- and SCFA-pretreated, EMCV-infected WT or anti-IFNAR1 IgG treated mice (*n* = 5). Plotted data represent the mean ± s.d. Unpaired two-tailed Student’s *t* tests were used for comparison of means between treatment groups. *p < 0.05; **p < 0.01; ***p < 0.001; ****p < 0.0001; ns not significant.

To determine whether acetate and propionate have a direct antiviral effect on macrophages, BMDMs were treated with SCFAs at different concentrations for 24 h before MNV-1 infection. Indeed, acetate or propionate pretreatment before MNV-1 infection significantly impaired viral replication in BMDMs in a dose-dependent manner, whereas butyrate did not affect MNV-1 growth whatsoever (Fig. 3a and Supplementary Fig. S5A). A similar protective pattern was observed in RAW264.7 cells treated with acetate or propionate prior to EMCV infection (Supplementary Fig. S5B).

To define how acetate and propionate mediate antiviral macrophage functions against MNV-1, we performed transcriptional profiling of infected BMDMs with or without acetate, propionate, butyrate or lactate pretreatment. At 8 h post-infection (hpi), unbiased hierarchical clustering and GO analyses indicated that IFN-I signaling was the most significantly enriched pathway in acetate- and propionate-pretreated BMDMs compared with butyrate-, lactate- or vehicle-pretreated cells (Fig. 3b and Supplementary Fig. S5C). Consistent with this, pretreatment with acetate or propionate but not butyrate or lactate led to significantly higher IFN-β expression in BMDMs, at both the transcriptional and translational level (Fig. 3c). However, there was no apparent IFN-β upregulation in uninfected BMDMs pretreated with acetate or propionate (Supplementary Fig. S5D). These data, concordant with the result of transcriptome analysis, suggest that the antiviral, but not basal, transcriptional IFN-I response program of macrophages is altered by acetate and propionate. We also evaluated the expression of proinflammatory cytokines including IL-1β, IL-6 and TNF-α using qRT-PCR and found increased levels of cytokine transcripts in infected BMDMs pretreated with acetate or propionate compared with those receiving butyrate, lactate or untreated cells (Supplementary Fig. S5E), indicating that acetate and propionate pretreatment caused a generally enhanced innate antiviral immune response against MNV-1 infection.

Consistent with the IFN-β analysis, there was a marked upregulation of IFN-stimulated genes (ISGs) including *Isg15*, *Isg56*, *Oas1a* and *Mx1* in the BMDMs pretreated with acetate or propionate compared with those receiving butyrate or lactate (Fig. 3d). Further, EMCV-stimulated BMDMs exhibited a significant increase in IFN-β expression in the presence of acetate or propionate (Supplementary Fig. S5F). We also measured MNV-1 replication in BMDMs isolated from *Irf3*^*−/−*^, *Ifnar*^*−/−*^ and *Stat1*^*−/−*^ mice and observed no differences in viral titers regardless of SCFA treatment (Fig. 3e), demonstrating that inhibition of MNV-1 by acetate and propionate depends on important signaling molecules in the IFN-I induction pathway and amplification loop.

We sought to confirm the role of acetate and propionate in enhancing IFN-I production and restricting MNV-1 infection in macrophages at mucosal sites and found increased expression of IFN-β and decreased viral titers in the PP macrophages of MNV-1-infected, Van-treated mice administrated with acetate or propionate compared to vehicle- or butyrate-treated controls at 1 dpi (Fig. 3f, g). However, the effects of acetate or propionate on enhancement of IFN-β expression and reduction of MNV-1 replication were diminished in Van-treated *Ifnar*^*−/−*^ mice (Fig. 3f, g). In support of this finding, we observed that Rg3 administration increased IFN-β expression in the PPs of MNV-1-infected WT B6 mice, while Van treatment eliminated this phenotype (Supplementary Fig. S6A, B). Correspondingly, colonization of *B. coccoides* or *B. obeum* reestablished IFN-I responses and control of viral replication in macrophages isolated from PPs of MNV-1 infected, Van-treated WT B6 mice at 1 dpi, whereas the effects of *Blautia* colonization were diminished in Van-treated *Ifnar*^*−/−*^ mice (Supplementary Fig. S6C, D).

Since EMCV infection overwhelms the defenses of *Ifnar*^−/−^ mice [15], we injected Van-treated WT B6 mice with an anti-IFNAR1 antibody [18] to evaluate the role of IFN-I signaling in acetate/propionate-driven protection against systemic EMCV infection. Protection against lethal systemic EMCV infection conferred by acetate/propionate supplementation (Fig. 2f) in Van-treated mice was diminished when these animals were administrated with the IFNAR1-blocking antibody (Fig. 3h). Acetate/propionate supplementation also caused elevated expression of IFN-β in splenic macrophages of EMCV-infected, Van-treated WT B6 mice at 3 dpi, but failed to do so in Van-treated mice administrated with the IFNAR1-blocking antibody (Fig. 3i). Supporting these data, we observed no differences in mortality rate between IFNAR1-blocked mice regardless of Rg3 treatment, whereas WT mice given a nonspecific control IgG showed a significantly higher survival rate after treatment with Rg3 compared with vehicle control (Supplementary Fig. S6E). Moreover, elevated expression of IFN-β was exhibited in splenic macrophages of Rg3- versus vehicle-treated WT B6 mice following EMCV infection, an effect that was neutralized by Van pretreatment (Supplementary Fig. S6F).

Consistently, the protective effect exerted by *B. coccoides* or *B. obeum* colonization (Fig. 1d) was abolished in Van-treated mice administered the IFNAR1-blocking antibody (Supplementary Fig. S6G). Furthermore, IFN-β expression was restored in the splenic macrophages of EMCV-infected, Van-treated WT mice, but not those received IFNAR1-blocking antibody following *Blautia* spp. colonization at 3 dpi (Supplementary Fig. S6H). Taken together, these results indicate that acetate and propionate, which are increased after Rg3 administration and *Blautia* spp. colonization, restrict both local and systemic infection of enteric viruses in an IFN-I signaling-dependent manner.

### Stimulation of IFN-I response in macrophages by acetate or propionate requires GPR43 signaling

**Fig. 4:**
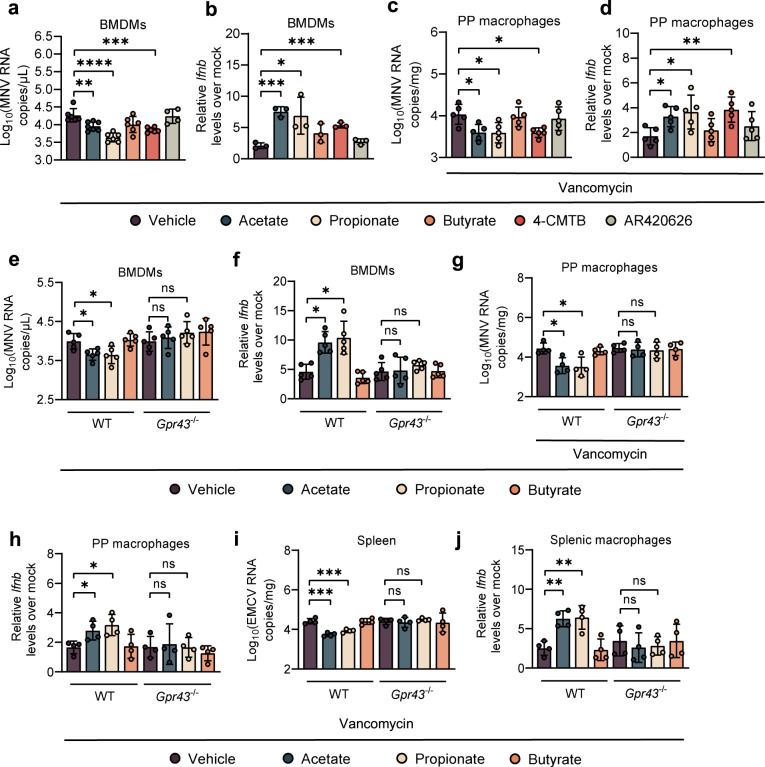
Acetate and propionate stimulate IFN-I responses by GPR43 signaling. Viral titers (**a**) at 24 hpi and relative *Ifnb* expression (**b**) at 8 hpi in WT BMDMs pretreated with acetate, propionate, butyrate, or GPR43 and GPR41 agonists (4-CMTB and AR420626) and subsequently infected with MNV (**a**, *n* = 6; **b**, *n* = 3). Viral titers (**c**) and relative *Ifnb* expression (**d**) at 1 dpi in PP macrophages sorted from Van- and SCFA-, 4-CMTB- or AR420626-pretreated, MNV-infected mice (*n* = 5). Viral titers (**e**) at 24 hpi and relative *Ifnb* expression (**f**) at 8 hpi in Van- and SCFA-pretreated, MNV-infected BMDMs (*n* = 5). Viral titers (**g**) and relative *Ifnb* expression (**h**) at 1 dpi in PP macrophages sorted from Van- and SCFA-pretreated, MNV-infected WT or *Gpr43*^−/−^ mice (*n* = 4). Viral titers (**i**) in splenocytes and *Ifnb* levels (**j**) in splenic macrophages collected at 3 dpi from Van- and SCFA-treated, EMCV-infected WT or *Gpr43*^−/−^ mice (*n* = 4). Plotted data represent the mean ± s.d. Unpaired two-tailed Student’s *t* tests were used for comparison of means between treatment groups. *p < 0.05; **p < 0.01; ***p < 0.001; ****p < 0.0001; ns not significant.

SCFAs can exert their functions via binding to GPR43 and GPR41 [19], and it has been shown that GPR43 is essential for acetate-induced IFN-I responses in pulmonary epithelial cells during RSV infection [9]. Considering this, we repeated the in vitro experiments in Fig. 3a using a GPR43 agonist (4-CMTB) or a GPR41 agonist (AR420626). We found that 4-CMTB, but not AR420626, caused the same pattern of response observed with acetate or propionate in MNV-1-infected BMDMs, reflected by reduced virus load and upregulated IFN-β (Fig. 4a, b). Additionally, 4-CMTB protected the WT B6 mice against MNV-1 infection by reducing the viral burden and augmenting IFN-β in PP macrophages at 1 dpi (Fig. 4c, d).

To elucidate the dependence of acetate/propionate stimulation of antiviral IFN-I on one of these receptors, we extracted BMDMs from WT B6 and GPR43 knockout (*Gpr43*^*−/−*^) mice, followed by acetate/propionate treatment and MNV-1 infection. Acetate/propionate pretreatment failed to reduce viral load or augment IFN-β expression in MNV-1-infected BMDMs extracted from *Gpr43*^*−/−*^ mice compared with WT B6 mice (Fig. 4e, f). Butyrate treatment had no effect on MNV-1 replication or IFN-I responses in BMDMs regardless of genotype (Fig. 4a–d). Similarly, siRNA knockdown of *Gpr43*, but not *Gpr41* significantly impaired MNV-1 replication and augmented IFN-β expression by the acetate/propionate treatment in WT B6 BMDMs (Supplementary Fig. S7A–C).

To further investigate the role of GPR43 in acetate/propionate-induced IFN-I responses against MNV-1 infection in vivo, Van-treated WT and *Gpr43*^*−/−*^ mice were administrated with acetate or propionate and then inoculated with MNV-1. No differences in viral titers and IFN-β levels were seen in PP macrophages from MNV-1-infected, Van-treated *Gpr43*^*−/−*^ mice, regardless of SCFA treatment (Fig. 4g, h).

Moreover, EMCV stimulation of acetate/propionate-pretreated BMDMs isolated from *Gpr43*^*−/−*^ mice failed to upregulate IFN-β expression compared with WT B6 control BMDMs (Supplementary Fig. S7D). Similar to the results of MNV-1 in vivo study, there were no differences between the acetate/propionate-pretreated and control groups in *Gpr43*^*−/−*^ mice with respect to viral burden in the spleen or IFN-β expression in the splenic macrophages, reflecting abrogated antiviral effect-induced by acetate/propionate in the absence of GPR43 (Fig. 4i, j). Thus, specific interaction between acetate/propionate and GPR43-primed macrophage IFN-I responses to prevent both local and systemic enteric virus infection.

### Acetate and propionate promote antiviral IFN-I response by upregulating intracellular calcium influx and virus-induced mtDNA release in macrophages depending on GPR43 and MAVS

**Fig. 5:**
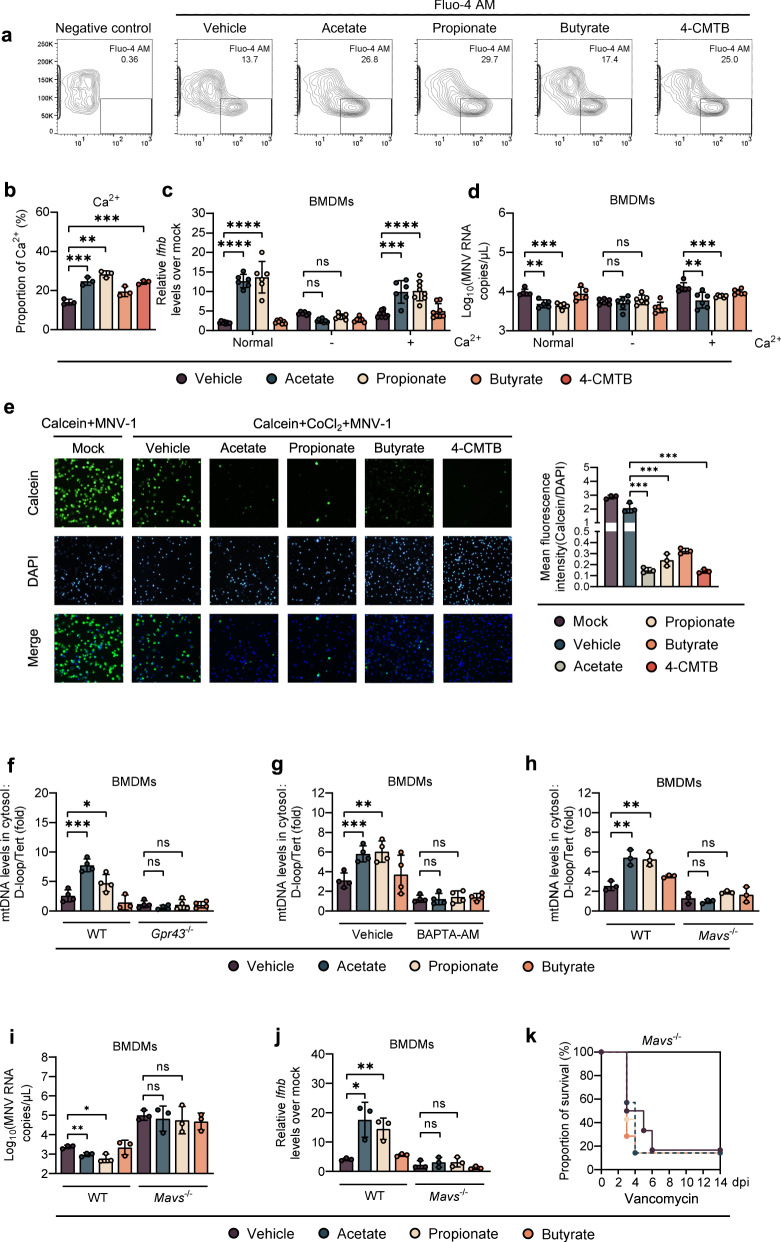
Acetate/propionate promote antiviral IFN-I responses by upregulating intracellular calcium influx and virus-induced mitochondrial DNA release. **a**, **b** Intracellular Ca^2+^ levels in WT BMDMs were measured after 24 h incubation with acetate, propionate, butyrate or 4-CMTB. **c**, **d** BMDMs were cultured in normal medium or calcium-free medium with or without supplementation with 0.2 g/L Ca^2+^. Relative *Ifnb* expression (**c**) at 8 hpi or viral titers (**d**) at 24 hpi in SCFA-pretreated, MNV-infected WT BMDMs were analyzed (*n* = 6). **e** The openness of mitochondrial permeability transition pore (mPTP) was determined at 24 hpi by mPTP kit in SCFA- and 4-CMTB-pretreated, MNV-infected BMDMs. **f** SCFA-pretreated WT or *Gpr43*^−/−^ BMDMs were infected with MNV for 24 h and cytosolic mtDNA was assessed by quantitative PCR (*n* = 4). **g** WT BMDMs were treated with 30 µM BAPTA-AM for 24 h, followed by 24 h incubation with SCFAs prior to MNV infection. Cytosolic mtDNA levels were analyzed at 24 hpi (*n* = 4). **h** Cytosolic mtDNA levels were measured at 24 hpi in WT or *Mavs*^−/−^ BMDMs pretreated with acetate, propionate, or butyrate and infected with MNV (*n* = 3). Viral titers (**i**) at 24 hpi and *Ifnb* levels (**j**) at 8 hpi in SCFA-pretreated, MNV-infected WT or *Mavs*^−/−^ BMDMs (*n* = 3). **k** Groups of differently treated *Mavs*^−/−^ mice were i.p. infected with EMCV, and survival curves were documented (*n* = 6–7). Plotted data represent the mean ± s.d. Unpaired two-tailed Student’s *t* tests were used for comparison of means between treatment groups. *p < 0.05; **p < 0.01; ***p < 0.001; ****p < 0.0001, ns not significant.

Acetate stimulates GPR43 and induces intracellular calcium influx in myotube cells [20] and it has been shown that IFN-I response is connected to the intracellular Ca^2+^ levels and can be suppressed in the presence of Ca^2+^ chelators [21]. Thus, we pretreated BMDMs isolated from WT B6 mice with SCFAs and 4-CMTB. As predicted, intracellular Ca^2+^ levels were significantly induced by acetate, propionate, or 4-CMTB, but not by butyrate treatment (Fig. 5a, b). However, this effect was completely abolished in BMDMs collected from *Gpr43*^*−/−*^ mice (Supplementary Fig. S8A). WT BMDMs primed by acetate/propionate had significantly lower IFN-β levels and higher MNV-1 titers when cultured with Ca^2+^-free cell culture medium, an outcome reversed by addition of Ca^2+^ (Fig. 5c, d). Similarly, treatment of WT BMDMs with a cell-permeable Ca^2+^ chelator BAPTA-AM prevented the IFN-β upregulation and MNV-1 inhibition resulting from acetate/propionate treatment (Supplementary Fig. S8B, C). Thus, enhanced IFN-I production and MNV-1 inhibition was mediated by intracellular calcium influx in macrophages, which was modulated by GPR43 signaling.

Changes in cytosolic Ca^2+^ causes a mitochondrial permeability transition, which leads to the leakage of mtDNA into the cytoplasm [22]. Thus, we hypothesized that changes of intracellular calcium influx caused by GPR43 activation enhance mtDNA leakage after enteric virus infection. To test this, we first evaluated the degree of mitochondrial permeability transition pore (mPTP) opening using a specific kit. We observed a significantly higher degree of mPTP opening (dimmer green fluorescence) in acetate-, propionate- or 4-CMTB-treated BMDMs compared to vehicle- or butyrate-treated BMDMs upon MNV-1 infection (Fig. 5e). However, this effect was diminished by treatment with BAPTA-AM (Supplementary Fig. S8D). Consistently, we found that cytosolic mtDNA release was triggered by MNV-1 infection in WT BMDMs (3-fold higher compared to uninfected cells), and was significantly enhanced by pretreatment with acetate or propionate but not butyrate; this effect was completely abrogated by GPR43 depletion or BAPTA-AM treatment (Fig. 5f, g).

As it has been reported that mtDNA release during IAV infection requires activation of MAVS-dependent signals [23], we next examined whether MAVS is required for acetate/propionate-enhanced mtDNA release triggered by MNV-1 infection. To this end, we infected WT or MAVS-deficient BMDMs with MNV-1 following acetate/propionate treatment. Indeed, we found that the acetate/propionate-enhanced, MNV-1 induced mtDNA release into the cytosol was abolished in BMDMs isolated from *Mavs*^−/−^ mice (Fig. 5h). In addition, Acetate/propionate pretreatment did not lead to descending viral load or ascending IFN-β expression in MNV-1-infected, MAVS-deficient BMDMs (Fig. 5i, j). Moreover, *Blautia* spp. colonization or acetate/propionate treatment in Van-treated *Mavs*^−/−^ mice conferred no protection against systemic EMCV infection (Fig. 5k and Supplementary Fig. S8E).

### Acetate and propionate enhance IFN-I response in macrophages and limit enteric virus infection through the GPR43-cGAS-STING signaling axis

**Fig. 6:**
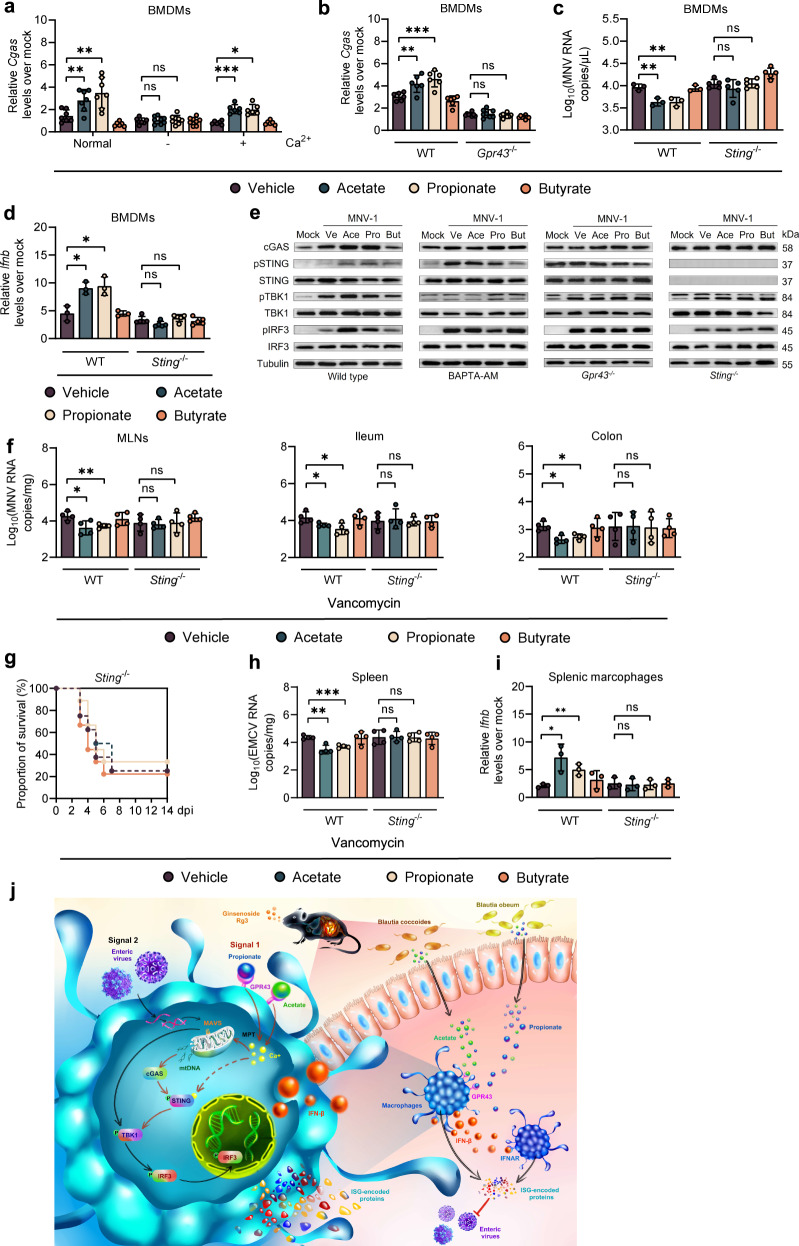
Acetate/propionate promote IFN-I responses in macrophages and limit enteric virus infection through the GPR43-cGAS-STING signaling axis. **a** Relative *Cgas* expression at 8 hpi in WT BMDMs cultured in normal medium or calcium-free medium with or without supplementation with 0.2 g/L Ca^2+^ pretreated with SCFAs and infected with MNV. **b** Relative *Cgas* expression at 8 hpi in BMDMs from WT or *Gpr43*^−/−^ mice pretreated with SCFAs and infected with MNV. Viral titers (**c**) at 24 hpi and *ifnb* levels (**d**) at 8 hpi in SCFA-pretreated, MNV-infected WT or *Sting*^−/−^ BMDMs (*n* = 3–5). **e** Western blot of cGAS, IRF3, pIRF3, STING, pSTING, TBK1, pTBK1 and tubulin in WT BMDMs with or without 30 µM BAPTA-AM, or BMDMs from *Gpr43*^−/−^ or *Sting*^−/−^ mice. **f** Viral burden analysis at 1 dpi in MLNs, ileum and colon tissues collected from WT or *Sting*^−/−^ mice pretreated for 2 weeks with 200 mM acetate, propionate or butyrate in drinking water and then infected with 2 × 10^7^ TCID_50_ MNV (*n* = 4). **g** Groups of Van-treated and 200 mM acetate-, propionate- or butyrate-pretreated WT or *Sting*^−/−^ mice were i.p. infected with 2 × 10^2^ TCID_50_ of EMCV, and survival curves were documented (*n* = 8). **h** Viral titers at 3 dpi of splenic tissue collected from SCFA-pretreated, EMCV-infected WT or *Sting*^−/−^ mice (*n* = 4). **i** *Ifnb* levels in splenic macrophages sorted at 3 dpi from Van- and SCFA-pretreated, EMCV-infected WT or *Sting*^−/−^ mice (*n* = 4). **j** Schematic illustration of how the *Blautia* spp.-associated metabolites acetate and propionate protect against enteric virus infection through the GPR43-cGAS-STING-IFN-I axis. Plotted data represent the mean ± s.d. Unpaired two-tailed Student’s *t* tests were used for comparison of means between treatment groups. *p < 0.05; **p <0.01; ***p < 0.001; ns not significant.

The importance of cytosolic mtDNA in guanosine monophosphate-adenosine monophosphate synthase (cGAS)- and stimulator of IFN genes (STING)-mediated IFN-I expression and innate immune defense against RNA viruses has been highlighted recently [23–25]. To this end, we then tested whether the acetate/propionate-induced intracellular calcium influx triggered by GPR43 activation activated cGAS in MNV-1-infected BMDMs. As expected, acetate/propionate stimulation showed no effect on enhancement of cGAS expression upon MNV-1 infection when the WT BMDMs were cultured in Ca^2+^-free cell culture medium, while Ca^2+^ supplementation reestablished this effect (Fig. 6a). A similar pattern of reduction of cGAS expression was observed when BAPTA-AM was used on WT BMDMs (Supplementary Fig. S8F). Consistently, the acetate/propionate-induced increase in cGAS expression after MNV-1 infection was dependent on GPR43 (Fig. 6b and Supplementary Fig. S8G). Moreover, acetate/propionate pretreatment did not affect viral replication or IFN-β expression in BMDMs treated with cGAS or STING siRNA or in BMDMs isolated from *Sting*^−/−^ mice (Fig. 6c, d and Supplementary Fig. S8H, I). Furthermore, we demonstrated that pretreatment with acetate or propionate but not butyrate markedly increased the level of cGAS, phosphorylated STING and IRF3 upon MNV-1 infection, differences that were lost in conditions of Ca^2+^ or GPR43 depletion (Fig. 6e).

To further confirm the requirement of STING for the promotion on IFN-I responses and restriction of enteric virus infection in vivo, we inoculated SCFA-pretreated *Sting*^−/−^ mice and WT controls with MNV-1 or EMCV. Consistent with our in vitro studies, an equivalent viral load was seen in the intestines and MLNs between SCFA-pretreated WT and *Sting*^−/−^ mice after MNV-1 infection (Fig. 6f). Likewise, the significant improvement in survival rate and decreased viral load in splenocytes induced by acetate/propionate treatment was completely lost in *Sting*^−/−^ mice inoculated with EMCV (Fig. 6g, h). STING depletion fully abrogated the enhancement of EMCV-induced IFN-β expression following SCFA administration in macrophages isolated from WT mice (Fig. 6i).

## Discussion

Recently, we reported that *Blautia coccoides* can restrict systemic EMCV infection by priming the IFN-I responses in peripheral mononuclear phagocytes [15]. Here, we further described how Rg3 enriches the gut commensal bacteria (*Blautia* spp.), whose production of acetate/propionate promotes the host antiviral IFN-I responses in macrophages to limit local and systemic enteric virus infection via the GPR43-cGAS-STING axis (Fig. 6j).

Ginsenosides like Rg3 represent the principle active pharmacological components of ginseng, the most well-known herbal medicine in Asian countries including China, Korea and Japan. Several studies have provided evidence of their in vitro inhibition of viral replication [17, 26], and modulation of gut microbiota was demonstrated as one of the mechanisms underlying the efficacy of ginsenosides [27]. In the current study, Rg3 treatment dramatically shifted the gut microbiome composition of mice, with significant increases in *Blautia* and *Faecalibaculum* spp. (Fig. 1b). Our findings are consistent with the report from Bai et al. that ginsenoside Rk3 intake led to a notable enrichment of *Bacteroide*s, *Alloprevotella*, *Blautia* and *Parabacteroides* spp. and increased SCFA levels in the gut [27].

It has been documented that most of the *Lachnospiraceae* and particularly *Blautia* are able to generate high levels of SCFAs, especially acetate and propionate [28]. The acetogenic *B. coccoides* produces acetate from acetyl-P_i_ by an acetate kinase encoded by the gene *ackA* [29]. The other representative *Blautia* strain, *B. obeum*, contains the gene *pduP*, which encodes a CoA-dependent propionaldehyde dehydrogenase that produces high levels of propionate from dexoy-sugars (rhamnose and fucose) via a propanediol pathway [30]. In future studies, we plan to construct respective mutants with silenced *ackA* and *pduP* genes to further correlate *Blautia* strains and SCFA production, which would enable us to mechanistically dissect the specific role of *Blautia*-derived SCFAs for limiting enteric virus infections in vivo.

A growing body of literature supports the role of gut microbiome-derived acetate in shaping host innate antiviral immunity at distant sites following infection with respiratory viruses such as IAV or RSV [9, 10, 31]. Enhanced innate immunity was linked to activated NOD-like receptor, pyrin domain-containing 3 (NLRP3) or IFN-I responses both in macrophages and pulmonary epithelial cells [9, 10]. For the first time, we described that *Blautia*-derived acetate and propionate induce IFN-I responses in macrophages both at mucosal and extraintestinal sites, conferring protection against self-limiting (MNV-1) and systemic (EMCV) enteric virus infections. Consistent with most of the aforementioned studies, the current work demonstrates that exogenous acetate/propionate induce antiviral IFN-I responses by activating GPR43 in macrophages. Although GPR41 and GPR43 are both SCFA receptors, propionate has the highest affinity for GPR43 and acetate is more selective for GPR43, whereas butyrate is more active on GPR41 [32]. This preference may explain our observation that acetate/propionate-mediated inhibition of MNV-1 replication was dependent on GPR43 but not GPR41 (Fig. 4). Although its primary targets are leukocytes, MNV can also infect intestinal epithelial cells (IECs) especially tuft cells [33]. It is unclear whether acetate/propionate also stimulate GPR43 in IECs to restrict MNV-1 infection.

While a role for acetate/propionate in promoting IFN-I responses via GPR43 is established, the downstream signaling pathways involved are less well defined. Our study demonstrates that GPR43 is coupled to intracellular Ca^2+^ release and MAVS-dependent mtDNA release, which is generally consistent with a very recent study demonstrating that microbiota-derived acetate promotes oligomerization and signaling of MAVS through GPR43 activation and NLRP3 engagement, leading to augmented IFN-I production against IAV [10]. These studies are mutually supportive because intracellular Ca^2+^ and mtDNA release are both damage-associated molecular patterns (DAMPs) that can be recognized by NLRP3 [34]. Also, both studies demonstrate that MAVS is required for acetate-GPR43 enhancement of virus-induced IFN-I. Acetate or propionate treatment alone did not induce detectable IFN-I responses prior to infection in BMDMs (Fig. 3b and Supplementary Fig. S4D), suggesting that endogenous microbiota-derived SCFA signaling through GPR43 is not sufficient to activate MAVS, but requires a second signal during viral infection to fully activate IFN-I pathways. This delicately balanced sensory system is necessary to maintain a poised basal state of immune cells to fight viral infection and at the same time avoid autoimmunity resulting from excessive IFN-I expression.

Few studies have shown that the microbiota impacts IFN responses through the cGAS-STING pathway. Gutierrez-Merino et al. reported that beneficial commensal lactic acid bacteria can trigger IFN-I production via direct recognition by STING and MAVS in vitro, however no analysis was performed in vivo [35]. A recent study conducted by Erttmann et al. suggested that the gut microbiota induces a systemic IFN-I response against both DNA and RNA viruses through cGAS-STING signaling via membrane vesicle-mediated delivery of bacterial DNA. Their work identified a mechanism by which the microbiota instructs cGAS-STING-IFN-I axis independently of bacterial metabolites.

In summary, our work identified an herbal monomer, Rg3, to be protective against enteric virus infections locally and systemically by enriching acetate/propionate-producing commensal bacteria in the gut, which induced host IFN-I responses through the GPR43-cGAS-STING axis. These findings build on the limited understanding of SCFA-GPR43 activation of IFN-I signaling pathways, and the properties of Rg3 and *Blautia* spp. have a potential therapeutic value that may be applicable to other enteric virus infections such as human norovirus, enteric virus 71 and poliovirus.

## Materials and methods

### Viruses, bacteria and cell culture

Recombinant virus derived from a full-length clone of EMCV strain BJC3 (GenBank accession no. DQ464062) was used in this study [36]. Viral titers of EMCV stocks were determined by a standard TCID_50_ assay as described previously[37]. Stocks of recombinant MNV-1 (GenBank accession no. KC782764) were generated as previously described [38]. In brief, 293T cells were transfected with 5 µg of infectious clone plasmid DNA per 10^6^ cells using Lipofectamine 2000 (Life Technologies) and lysed by freeze–thawing 1 d post-transfection. 293T lysates were used to infect RAW264.7 cells at an MOI of 0.05. RAW264.7 lysates were prepared when ~90% of cells displayed cytopathic effect and were purified through a 25% sucrose cushion by centrifugation at 25,000 × *g* for 5 h. Viral stocks were titrated using a standard TCID_50_ assay [38].

*B. coccoides* was purchased from ATCC (ATCC 29236) and cultured in modified chopped-meat medium (ATCC medium 1490; ELITE-MEDIA) at 37 °C under anaerobic conditions. *B. obeum* was purchased from DSMZ (DSM 25238) and cultured in modified PYG medium (DSMZ medium 104) at 37 °C under anaerobic conditions. *C. butyricum* was purchased from ATCC (ATCC 19398) and cultured in thioglycolate medium (Hopebio) at 37 °C under anaerobic conditions. The concentration of each bacterial species was quantified based on the optical density at 600 nm (OD600).

RAW264.7 cells, 293T cells and BHK-21 cells were purchased from ATCC and cultured in Dulbecco’s modified Eagle medium (DMEM) supplemented with 10% fetal bovine serum (GIBCO, Invitrogen Corporation, Carlsbad, CA, USA), 100 U/mL penicillin, 100 µg/mL streptomycin at 37 °C and 5% CO_2_. BMDMs were isolated from bone marrow extracted from mouse femurs and tibiae as previously described [2]. Cells were cultured at 37 °C and 5% CO_2_ in DMEM supplemented with 20% FBS and 30% supernatant of filtered L929 cells, 100 U/mL penicillin, 100 µg/mL streptomycin at 37 °C and 5% CO_2_ for 7 days prior to the experimental procedure.

### Animals and viral infections

Mice were maintained in a specific-pathogen-free (SPF) facility with a temperature- and humidity-controlled environment (22 ± 2 °C, 50 ± 10% humidity), and all animal experiments were strictly carried out in accordance with protocol no. 117113 approved by the Institutional Animal Care and Use Committee of Zhejiang University. 6-to-8-week-old, sex-matched mice were used for all experiments. C57BL/6J wild-type mice and Rag knockout mice (*Rag*^−/−^) mice were purchased from the Model Animal Research Center of Nanjing University (Nanjing, China). IFN-I-receptor knockout mice (referred to as *Ifnar*^−/−^) were a kind gift from Dr. Yu Chen (Wuhan University, Hubei, China). IFN regulatory factor 3-deficient mice (referred to as *Irf3*^−/−^) mice were provided by Dr. Jin Jin (Zhejiang University, Hangzhou, China). Signal transducer and activator of transcription 1 (referred to as *Stat1*^−/−^) mice were kindly provided by Dr. Rongbin Zhou (University of Science and Technology of China, Hefei, China). Mitochondrial antiviral signaling protein knockout mice (referred to as *Mavs*^−/−^) were kindly provided by Dr. Jiyong Zhou (Zhejiang University, Hangzhou, China). Free fatty acid receptor 2 knockout mice (referred to as *Gpr43*^−/−^) and STING knockout mice (referred to as *Sting*^−/−^) were purchased from Cyagen Biosciences Inc (Suzhou, china).

For virulence assays, wild-type B6 mice were inoculated orally with 2 × 10^7^ TCID_50_ of MNV-1 CW3 in 25 μL inoculum or infected intraperitoneally with 1 × 10^3^ TCID_50_ of EMCV in 100 μL inoculum; *Ifnar*^−/−^ mice were inoculated orally with 5 × 10^3^ TCID_50_ of CW3 in 25 μl inoculum. For EMCV survival experiment, wild-type mice were infected intraperitoneally with 200 TCID_50_ of EMCV in 100 μL inoculum, *Mavs*^−/−^, *Rag*^−/−^ or wild-type mice that treated with anti-IFNAR1 IgG (1.5 mg) by intraperitoneal injection 1 day before infection, were infected intraperitoneally with 50 TCID_50_ of EMCV in 100 μL inoculum. Clinical symptoms were observed and documented blindly using the following scoring criteria: 0, normal; 1, hunched posture and trembling; 2, hind-limb paralysis; 3, dyspnea and lack of responsiveness to touch; 4, sudden death.

### Antibiotic treatment, FMT and bacterial colonization

For single antibiotic treatment, mice were given either ampicillin (Amp), neomycin (Neo), metronidazole (Metro), or vancomycin (Van) (167 mg/mL) daily for 5 days via oral gavage. After the fifth day of oral gavage, the same antibiotic was added to the drinking water at a concentration of 1 g/L for Amp, Neo, Metro or Van, respectively. Fecal samples collected from microbiota-depleted mice at the 5th day post-treatment were homogenized, plated on BHI agar with 10% sheep blood, and cultured under anaerobic conditions at 37 °C for 2 days followed by incubation under aerobic conditions at 37 °C for 1 day to confirm efficient microbial depletion. Animals were maintained with antibiotic- or PBS-containing water for the duration of the experiment.

For FMT experiments, 200 mg of pooled fecal pellets from wild-type mice were homogenized with sterile silica beads in 1.5 mL PBS at 45 Hz for 1 min and filtered with 70-mm strainers. Van-treated mice were subjected to gavage with 150 μL filtered stool homogenates individually [2].

For bacterial colonization experiments, Van-treated mice were subjected to gavage with 10^10^ CFU of *B. coccoides*, *B. obeum* or *C. butyricum* in 150 μL PBS on the 6th day after Van oral administration. At 48 h after FMT or bacterial colonization (on day 8 after oral Van gavage), stool samples were collected to determine the efficiency of colonization, and colonized mice were subsequently infected with MNV or EMCV at various MOIs.

### Animal protection studies

For the Rg3 (Biopurify) protection experiment, mice were given 50 mg/kg Rg3 daily for 3 days via oral gavage before MNV or EMCV infection. For the SCFA protection experiment, 200 mM acetate, propionate or butyrate (Sigma) was added to the drinking water for 2 weeks before infection, and kept in water for the rest of experiment. Van treatment was performed 1 week before infection. For sodium lactate (Sigma), mice were gavaged with 0.24 g/kg body weight sodium lactate three days before infection. For GPR43 and GPR41 agonist experiments, mice were treated intraperitoneally with 4-CMTB or AR420626 (10 mg/kg, MedChemExpress, China) for 24 h before MNV or EMCV infection.

### Mononuclear phagocyte depletion

Mononuclear phagocyte depletion experiments were performed as previously described (14). Briefly, animals were injected with 250 μL per mouse of clodronate liposomes (Yeasen) or control liposomes intraperitoneally on day 2 before Van treatment, on the day of Van treatment, and then on days 2 and 5 after Van treatment.

### Cell protection study

For the Rg3 protection experiment, BMDM or RAW264.7 cells were incubated with 0, 50, 100, 150, 200 μM Rg3 for 24 h and infected with MNV or EMCV (MOI = 0.05 or 5). For the SCFA protection experiment, 1 mM acetate, propionate, butyrate or 10 mM lactic acid (Sigma) was added to the medium for 24 h before MNV or EMCV infection. For respective GPR41, GPR43 and STING agonist or Ca^2+^ chelator treatments, 10 μM AR420626, 10 μM 4-CMTB, 10 μM DMXAA or 30 μM BAPTA-AM was added to the medium for 24 h before MNV or EMCV infection. Cell cultures were collected at 8 or 24 hpi for cytokine expression analysis or viral load quantification.

### Fecal bacteria quantification

Fecal bacteria were quantified by qRT-PCR after isolation of bacterial DNA using a TIANamp Stool DNA Kit (TIANGEN). qRT-PCR was performed using SYBR Green Real-time PCR Master Mix (TOYOBO) with primers listed in the Key Resources table.

### DNA extraction, 16S rRNA gene amplicon sequencing and data analyses

Fecal samples (~200 mg) were resuspended in Qiagen’s ASL buffer and homogenized for 2 min. Total fecal DNA was extracted from the supernatant using a QIAamp DNA Stool Mini Kit (Qiagen). DNA concentration and purity were measured by Qubit (Thermo Fisher Scientific). The stool DNA was then amplified using Phusion High-Fidelity PCR Master Mix (New England Biolabs) by PCR targeting the variable regions 3 and 4 (V3–V4) of the 16S rRNA gene. Multiplex sequencing of amplicons with sample-specific barcodes was performed using a NovaSeq System (Illumina). For sequence analysis, raw paired-end reads were quality-filtered by fastp and merged using usearch from further analysis. After removing chimeric sequences, sequences were clustered at a 97% similarity cutoff value to generate operational taxonomic units (OTUs) using UPARSE. For each representative sequence, the SILVA 138 database were used to annotate taxonomic information by usearch-sintax, with a 0.8 bootstrap confidence level. The abundance of bacterial species and their diversity were calculated based on OTU and taxonomic ranks.

### Viral load quantification

Tissue samples taken at different time points were harvested, weighed, and homogenized with stainless steel beads in 1 mL of DMEM supplemented with 2% fetal bovine serum (FBS) and titrated by qRT-PCR. Briefly, tissue samples were homogenized at 45 Hz for 1 min, and the homogenates were clarified by centrifugation at 5000 × *g* for 5 min. Total RNA was extracted with TRIzol reagent (Invitrogen) and subjected to qRT-PCR using one-step qRT-PCR kits (Toyobo) on an ABI 7500 Fast instrument. Primers and probe are listed in the Key Resources table.

### Cytokine expression analysis

Total RNA from bead-homogenized tissue samples or cell culture was extracted using TRIzol reagent (Invitrogen) following the manufacturer’s instructions. The cytokine level was determined using the HiScript II One Step qRT–PCR SYBR Green Kit (Vazyme), and normalized to GAPDH. Results are presented as fold change of cytokine expression in infected mice relative to that of mock animals ((2^−ΔΔ*CT*^); primers are listed in the Key Resources table.

IFN-β protein quantification was performed using a mouse IFN-β enzyme-linked immunosorbent assay (ELISA) kit (Multi Sciences) according to the manufacturer’s instructions.

### Flow cytometry

Splenocytes and PPs were harvested to analyze surface antigen levels on different cell subsets following blockade of Fcg receptors with anti-CD16/32. Fluorescently conjugated antibodies targeting CD3, CD4, CD8, CD19, CD45, CD64, CD80, CD11b, CD11c, F4/80, MHC-II, and NK1.1^+^ were used. Conventional dendritic cells (cDCs) were identified as F4/80^−^, CD11c^+^ and MHCII^+^. NK cells were identified as CD3^−^ and NK1.1^+^. Splenic macrophages were identified as CD45^+^, F4/80^+^ and CD11b^+^. Macrophages from PPs were identified as CD45^+^, CD11b^+^, CD64^+^, CD11c^int^ and MHCII^+^. CD4^+^ T-cells, CD8^+^ T-cells, and B-cells were identified as CD3^+^CD4^+^, CD3^+^CD8^+^, and CD19^+^ respectively. All samples were sorted using the BD FACSAria II SORP cell sorter. Sorted-macrophages were collected with 20% FBS DMEM for RNA extraction.

### siRNA transfection

siRNAs were transfected into BMDMs with Lipofectamine 3000 reagent (Invitrogen) following the manufacturer’s instructions. Mouse *Gpr41*, *Gpr43*, *Cgas*, *Sting* and negative control siRNAs were synthesized by GenePharma, with sequences listed in the Key Resources table. The efficiency of interference was determined by qRT-PCR.

### Intracellular calcium measurements

Intracellular calcium concentrations were measured by detecting the fluorescence of cells treated with a calcium-sensitive indicator, Fluo-4 AM (MedChemExpress, China). BMDMs harvested 7 days after differentiation were re-plated in 6-well plates (1 × 10^6^ cells/well) for 24 h. Subsequently, Ca^2+^ levels were determined with Fluo-4 AM using Synergy H1 (BioTek, US) or flow cytometry with a BD FACSVerse. Briefly, cells were washed twice with serum-free medium after re-plating. Then cells were incubated with 4 μM Fluo-4 AM for 30 min in darkness at 37 °C. After washing twice with serum-free medium, cell measurement was performed on a Synergy H1 instrument or a flow cytometer with an excitation band of 485/20 nm and fluorescence measured at 528/20 nm.

### Detection of mtDNA in cytosolic extracts

BMDMs were collected with a total of 1 mL buffer containing 150 mM NaCl, 50 mM HEPES pH 7.4, and 20 μg/mL digitonin (MedChemExpress, China) for measurement of cytosolic mtDNA release. The homogenates were incubated on an end-over-end rotator for 10 min. After centrifugation (1000 × *g*, 3 min) three times, the supernatants were transferred to fresh tubes and centrifuged at 20,000 × *g* for 10 min. Cytosolic DNA was isolated from these pure cytosolic fractions using FastPure Cell/Tissue DNA Isolation Mini Kit (Vazyme). qRT-PCR assays were used to detect the mtDNA levels (*D-loop*) in the cytosol relative to nuclear DNA levels (*Tert*) in the whole cell lysates, with primers listed in Key Resources table.

A mitochondrial permeability transition pore (mPTP) assay kit (C2009S, Beyotime) was used to detect the degree of mPTP opening. BMDMs were seeded into 24-well plates at 2 × 10^5^ cells/well. After processing, cells were incubated for 30 min with 500 μL/well fluorescence quenching solution, which was then replaced with pre-warmed DMEM for 30 min in darkness. Cell fluorescence was observed on a fluorescence microscope after washing twice with PBS.

### Transcriptomics analysis

BMDMs were pretreated with 1 mM acetate, propionate or butyrate for 24 h and infected with MNV (MOI = 1) for 8 h, then harvested for total RNA extraction with TRIzol reagent (Invitrogen). Samples were simultaneously assessed on an Agilent 4200 system (Agilent Technologies), Qubit 3.0 (Thermo Fisher Scientific) and Nanodrop One (Thermo Fisher Scientific). RNA-seq libraries were generated and sequenced by Guangdong Magigene Biotechnology. Triplicate samples of all assays were constructed in an independent library, and the following sequencing and analysis was performed: Whole messenger RNA-seq libraries were generated using a NEB Next Ultra Nondirectional RNA Library Prep Kit for Illumina (New England Biolabs) following the manufacturer’s recommendations. Clustering of the index-coded samples was performed on a cBot Cluster Generation System. After cluster generation, the library was sequenced on a NovaSeq 6000 System (Illumina) and 150-bp paired-end reads were generated. Raw data in fastq format were processed by Trimmomatic (v.0.36) to acquire clean reads, which were mapped to NCBI Rfam databases to remove the rRNA gene sequences using Bowtie2 (v.2.33). The remaining mRNA sequences were mapped to the reference genome by Hisat2 (2.1.0). HTSeq-count (v.0.9.1) was used to obtain the read count and function information of each gene according to mapping results. Differentially expressed genes (DEGs) of two conditions/groups were determined using edgeR (v.3.16.5). GO analysis of DEGs was implemented using clusterProfiler (v.3.4.4), in which gene-length bias was corrected.

### GC-MS/MS determination of fecal SCFA concentrations

20 mg of fecal samples was placed in a 2 mL EP tube, and 1 mL of phosphoric acid (0.5% v/v) solution and a small steel ball were added. Samples were ground uniformly, then vortexed for 10 min and ultrasonicated for 5 min. 100 μL of supernatant was collected after the mixture was centrifuged at 10,000 × *g* for 10 min at 4 °C. 0.5 mL MTBE (containing internal standard) solution was added, and the mixture was vortexed for 3 min and ultrasonicated for 5 min. After that, the mixture was centrifuged at 10,000 × *g* for 10 min at 4 °C, then the supernatant was collected and used for GC-MS/MS analysis.

For GC-MS/MS analysis of SCFAs, an Agilent 7890B gas chromatograph coupled to a 7000D mass spectrometer with a DB-FFAP column (30 m length × 0.25 mm i.d. × 0.25 μm film thickness, J&W Scientific, USA) was employed. Helium was used as carrier gas, at a flow rate of 1.2 mL/min. Injection was done in the split mode with an injection volume of 2 μL. The oven temperature was held at 90 °C for 1 min, raised to 100 °C at a rate of 25 °C/min, then raised to 150 °C at a rate of 20 °C/min, held for 36 s, raised to 200 °C at a rate of 25 °C/min, held for 0.5 min, after running for 3 min. All samples were analyzed in multiple reaction monitoring mode; the injector inlet and transfer line temperatures were 200 °C and 230 °C, respectively. SCFA content was detected by MetWare (http://www.metware.cn/) based on the Agilent 7890B-7000D GC-MS/MS platform.

### Statistics and reproducibility

Statistical analyses were performed with Prism GraphPad software v.8.0. Error bars represent standard error of the standard deviation (s.d.) in all figures, and *p* values were determined by two-tailed Student’s *t* test or analysis of variance. *R*^2^ was estimated for the correlation analysis of two continuous variables. A log-rank test was used for survival curves. A two-sided *p* value < 0.05 was considered statistically significant.

All experiments were repeated, with the number of replicates stated in the figure legends. Representative images for western blots were from at least 3 independent sample preparations.

### Supplementary information

Extended figure1

extended figure2

extended figure3

extended figure4

extended figure5

extended figure6

extended figure7

extended figure8

extended figure legends

extended tables

## Data Availability

16S rRNA sequence data are available in the Sequence Read Archive (SRA) under BioProject accession PRJNA986441.RNA-seq data are available in the SRA under BioProject accession PRJNA987146. And all the other data supporting the conclusions of this study is available in the paper and supplemental materials.
