# Correction to: Ginsenoside Rg3 enriches SCFA-producing commensal bacteria to confer protection against enteric viral infection via the cGAS-STING-type I IFN axis

**DOI:** 10.1093/ismejo/wraf123

**Published:** 2026-06-09

This is a correction to: Gan Wang, Jingtianyi Liu, Yanan Zhang, Jinyan Xie, Shuxian Chen, Yuhua Shi, Fushan Shi, Shu Jeffrey Zhu, Ginsenoside Rg3 enriches SCFA-producing commensal bacteria to confer protection against enteric viral infection via the cGAS-STING-type I IFN axis, *The ISME Journal*, Volume 17, Issue 12, December 2023, Pages 2426–2440, https://doi.org/10.1038/s41396-023-01541-7

In the original publication, the authors identified the fluorescence microscopy images for the butyrate-treated group in Figure 5e were erroneously reused from an independent replicate experiment due to duplicate file labeling (all misannotated as “Butyrate”) during figure assembly. This oversight was recently detected during archival verification of raw data.

The authors emphasize that this treatment group served solely as a negative control to validate the specificity of acetate/propionate in activating mitochondrial permeability transition pore (mPTP) opening. Its inclusion was designed to highlight the specific effects of acetate and propionate, and does not affect the study's central conclusion (ie, acetate/propionate-mediated regulation of type I interferon responses against enteric viral infection via mPTP modulation).


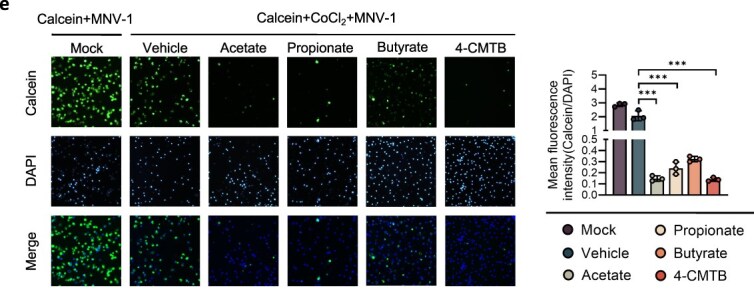


The authors sincerely regret the error and have implemented an automated image-data validation system in subsequent studies to prevent recurrence.

This error has been outlined only in this correction notice to preserve the version of record.

