# Supplementary figures and images for: Ginsenoside Rg3 enriches SCFA-producing commensal bacteria to confer protection against enteric viral infection via the cGAS-STING-type I IFN axis
Source: ISME J. 2023 Nov 10;17(12):2426–40. doi: 10.1038/s41396-023-01541-7 (PMC10689736; doi:10.1038/s41396-023-01541-7)

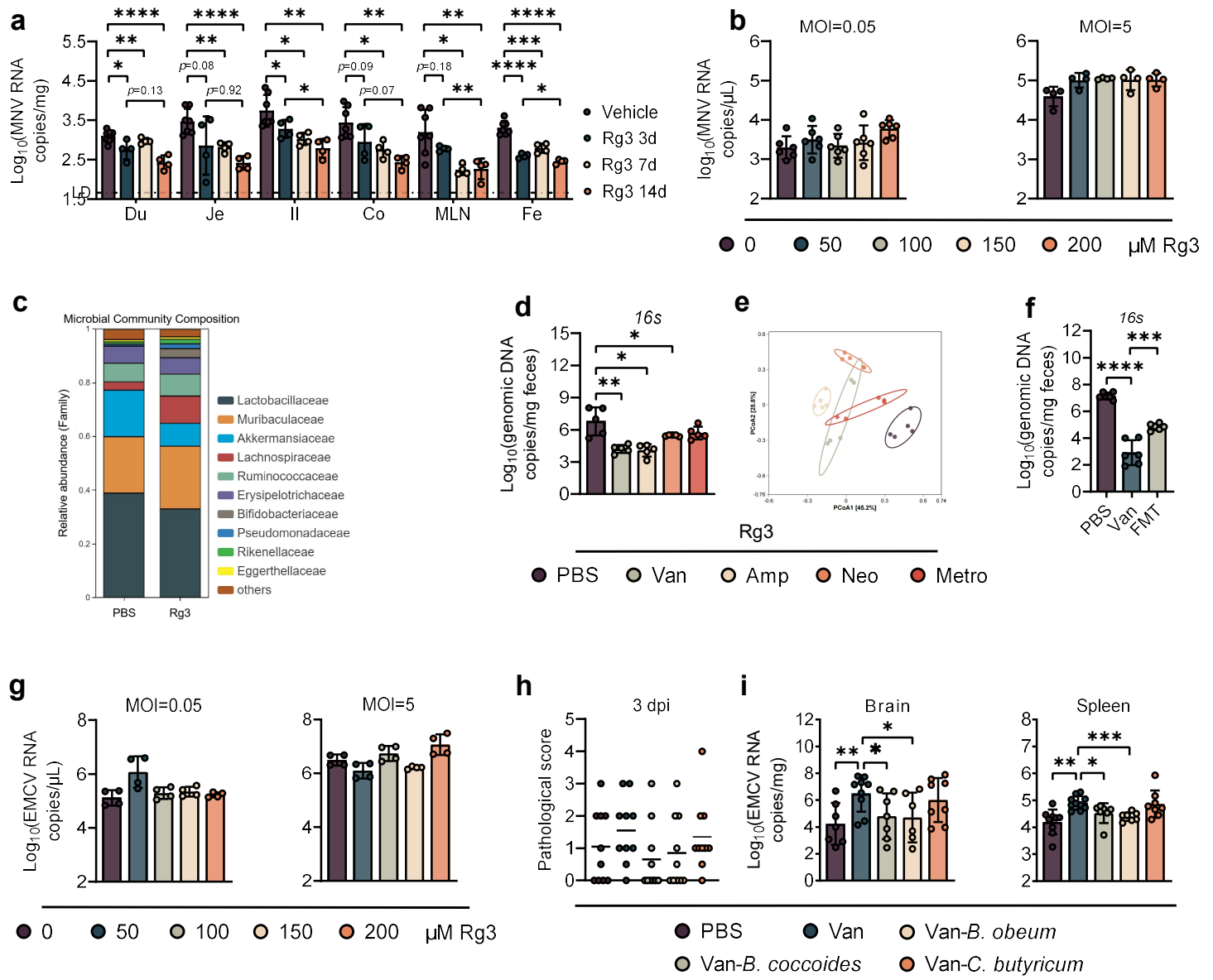

Supplement: Supplementary file 1 — Extended figure1 [file 41396_2023_1541_MOESM1_ESM.pdf]

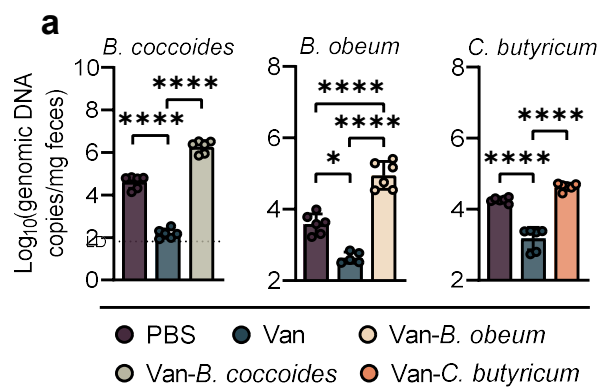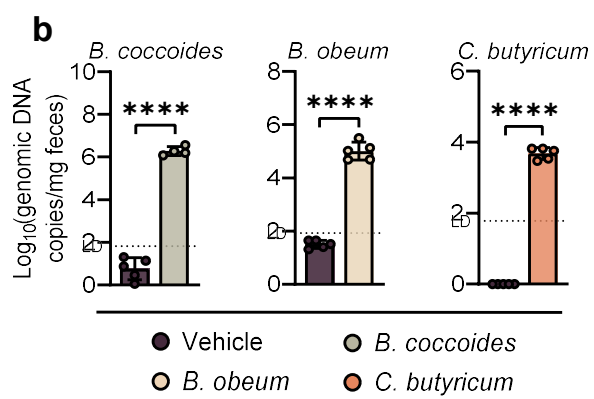

Supplement: Supplementary file 2 — extended figure2 [file 41396_2023_1541_MOESM2_ESM.pdf]

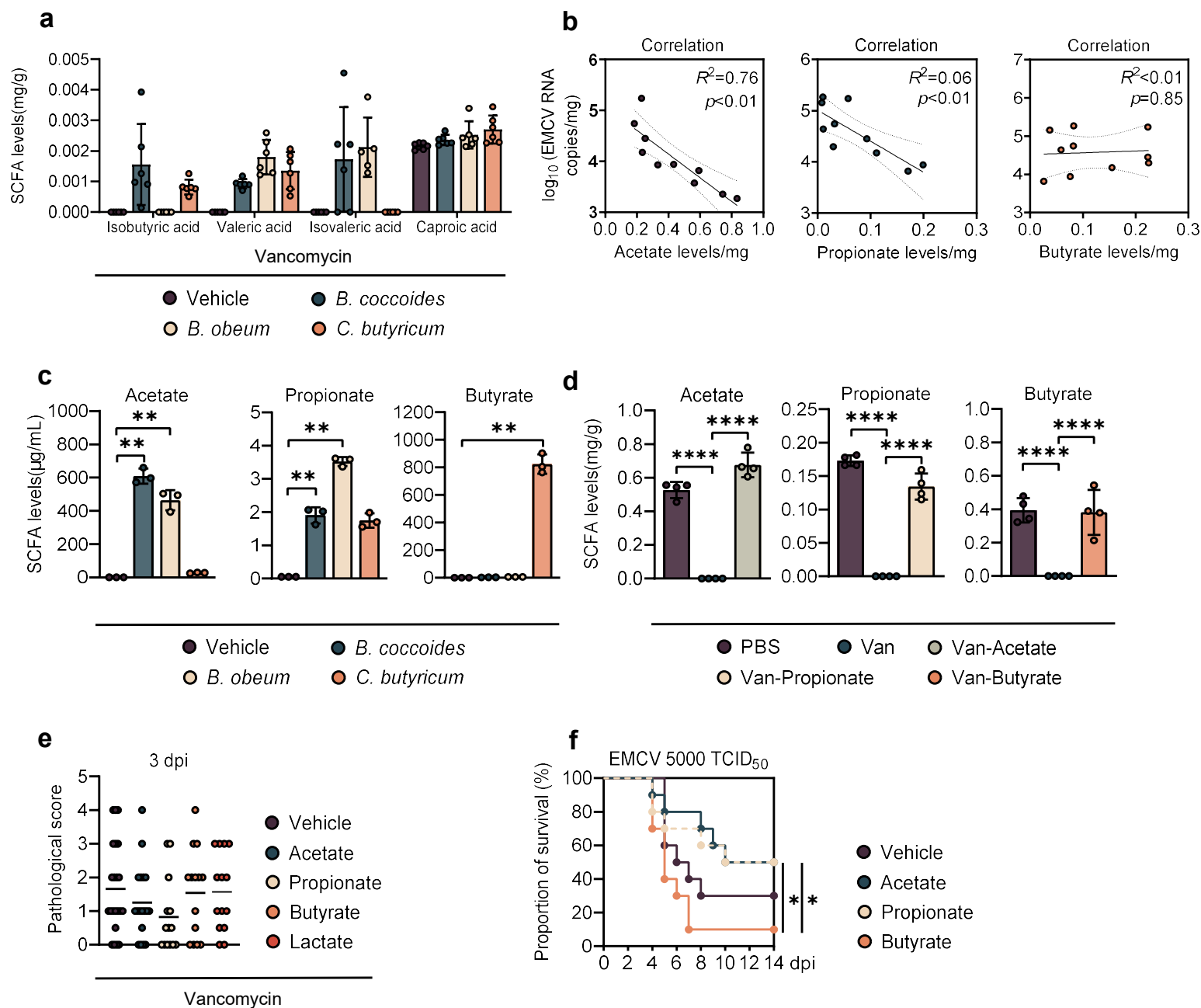

Supplement: Supplementary file 3 — extended figure3 [file 41396_2023_1541_MOESM3_ESM.pdf]

**a**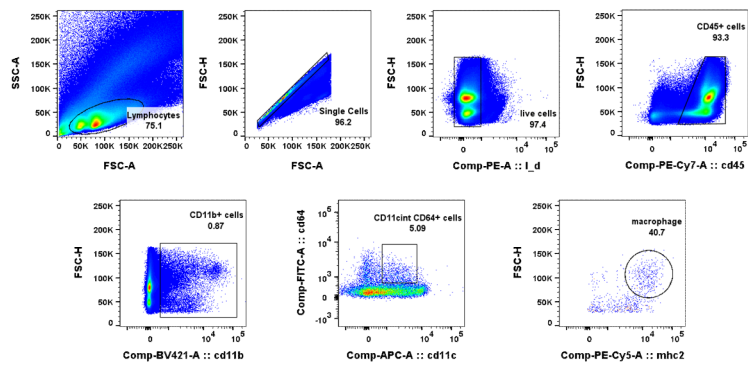**b**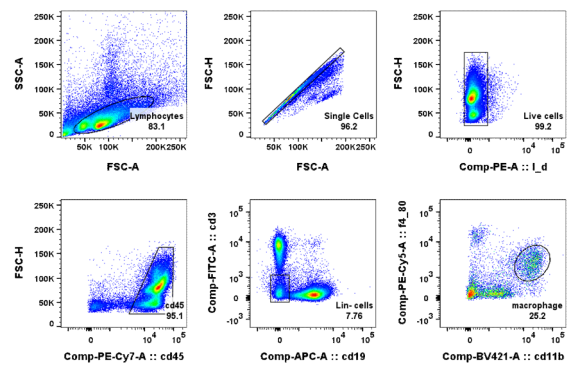**c**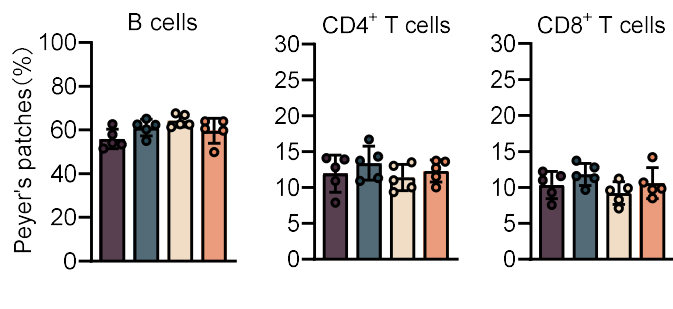**d**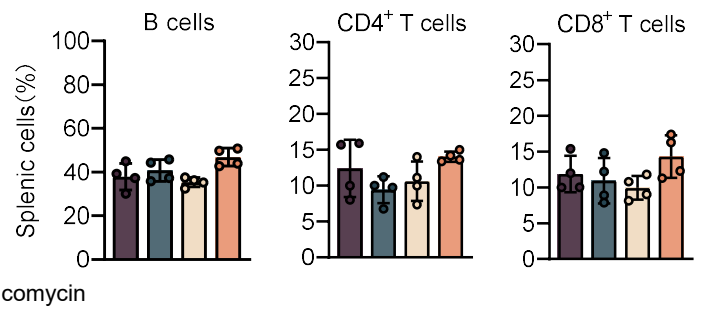**e**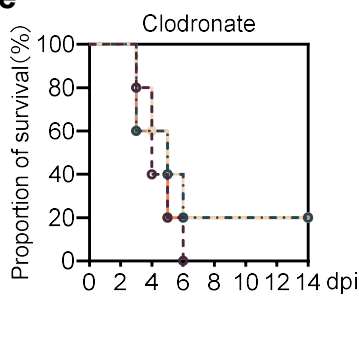**f**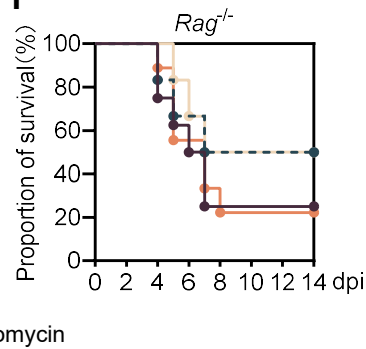

● Vehicle ● Acetate ● Propionate ● Butyrate

Supplement: Supplementary file 4 — extended figure4 [file 41396_2023_1541_MOESM4_ESM.pdf]

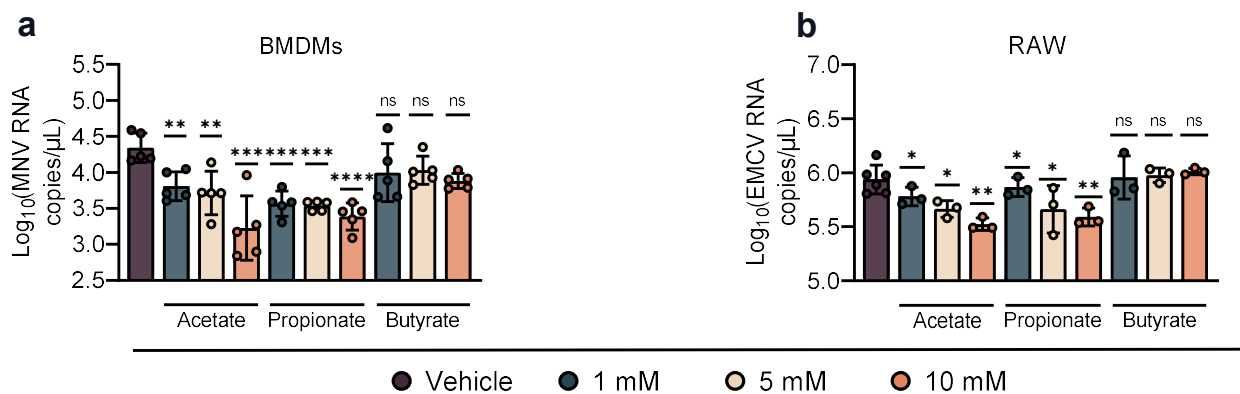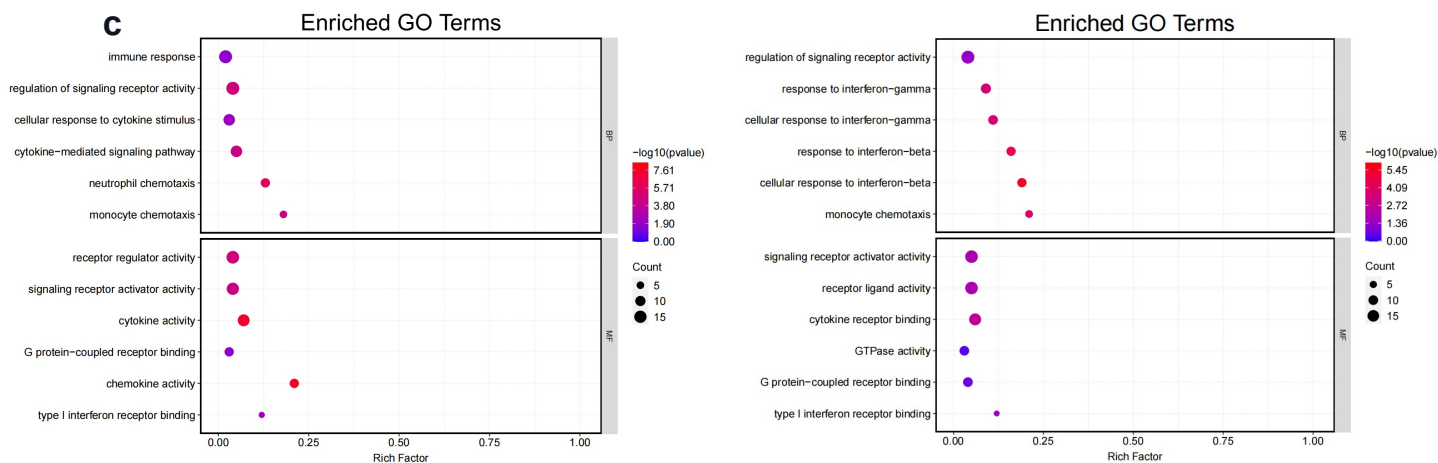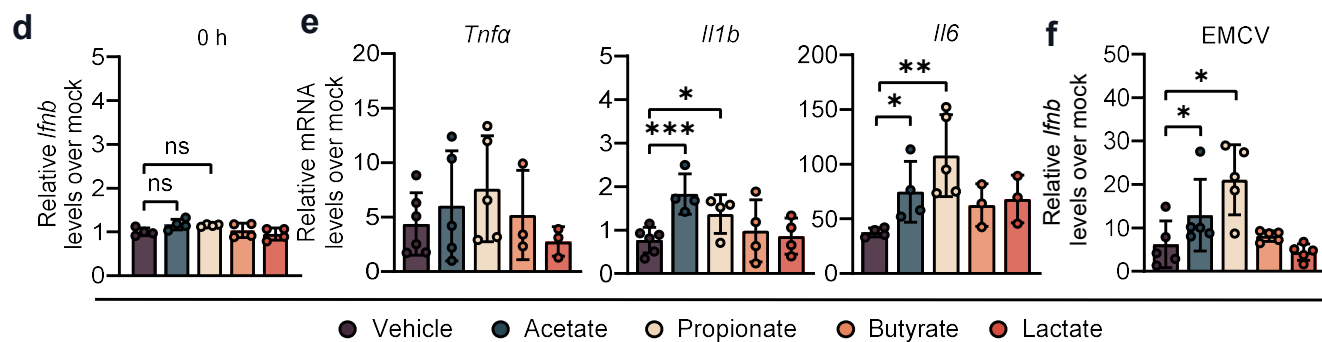

Supplement: Supplementary file 5 — extended figure5 [file 41396_2023_1541_MOESM5_ESM.pdf]

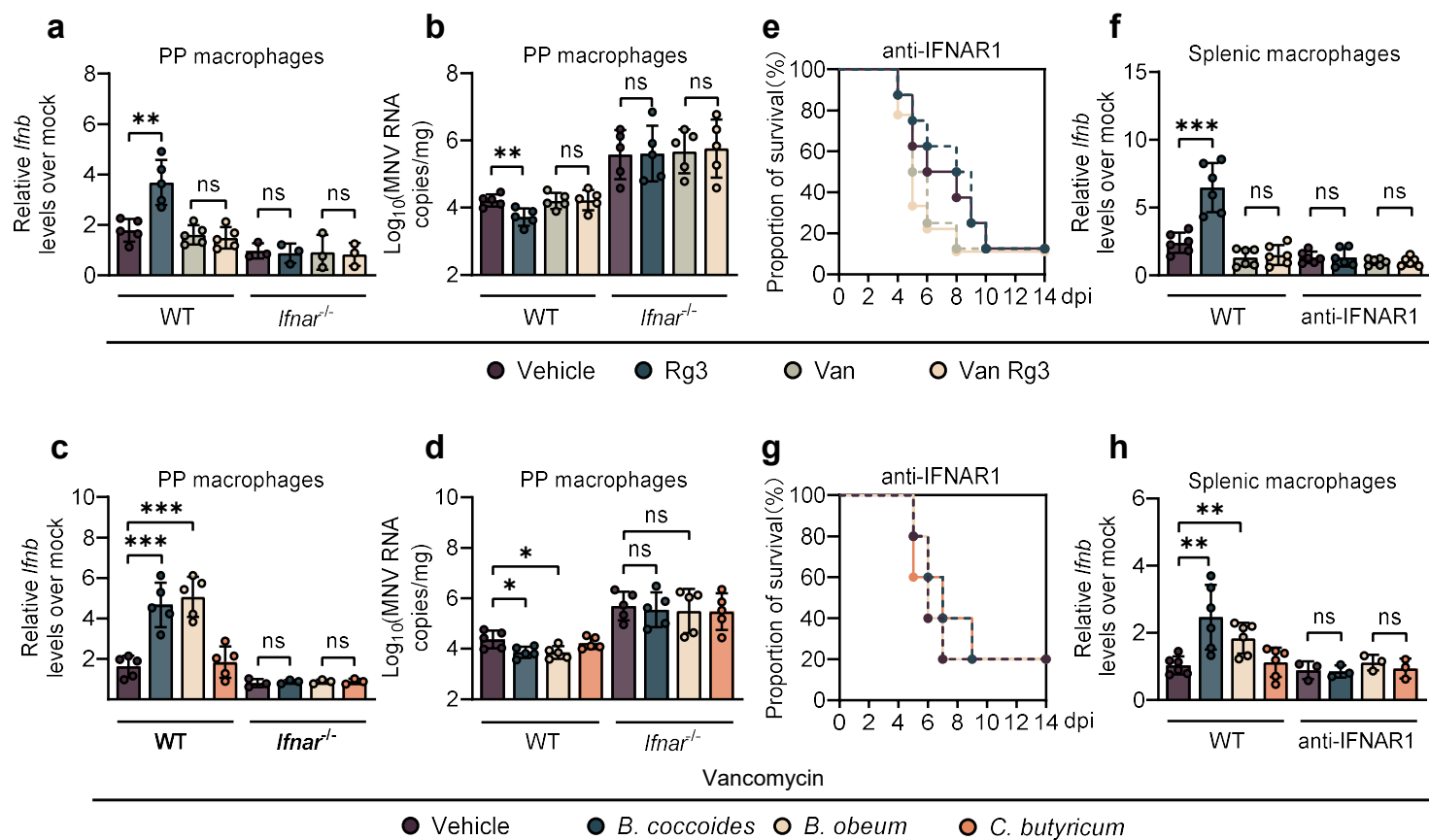

Supplement: Supplementary file 6 — extended figure6 [file 41396_2023_1541_MOESM6_ESM.pdf]

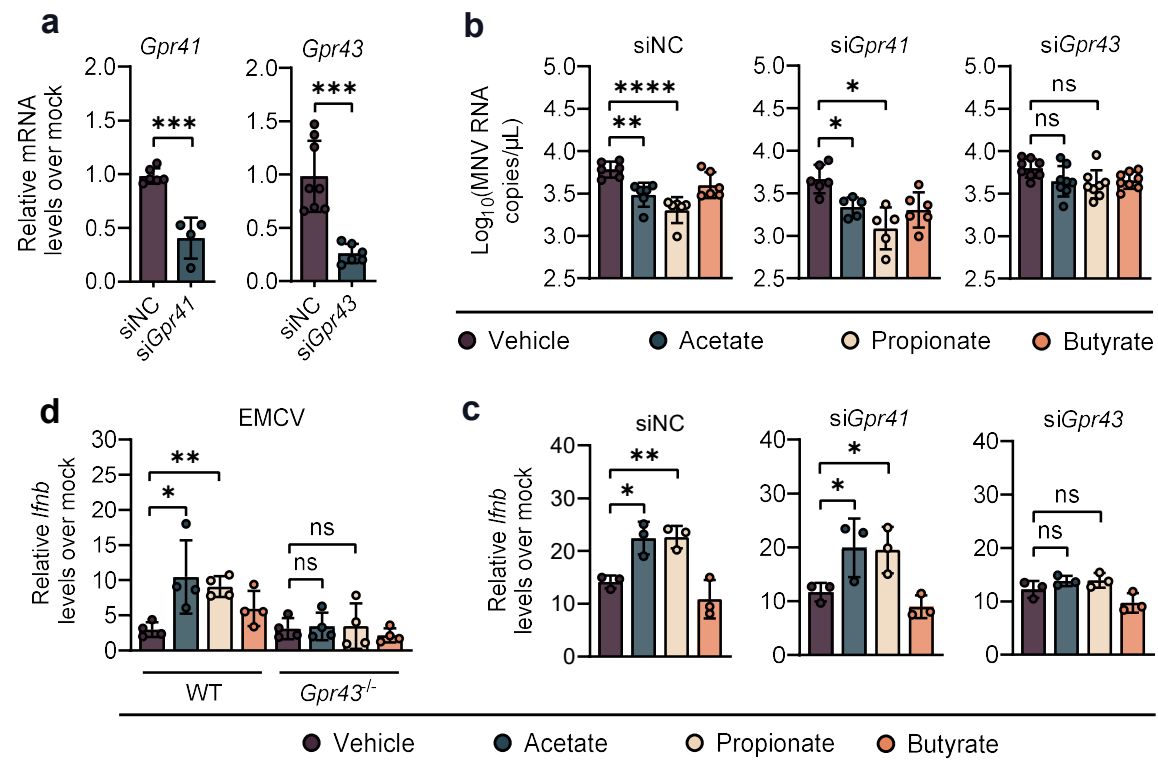

Supplement: Supplementary file 7 — extended figure7 [file 41396_2023_1541_MOESM7_ESM.pdf]

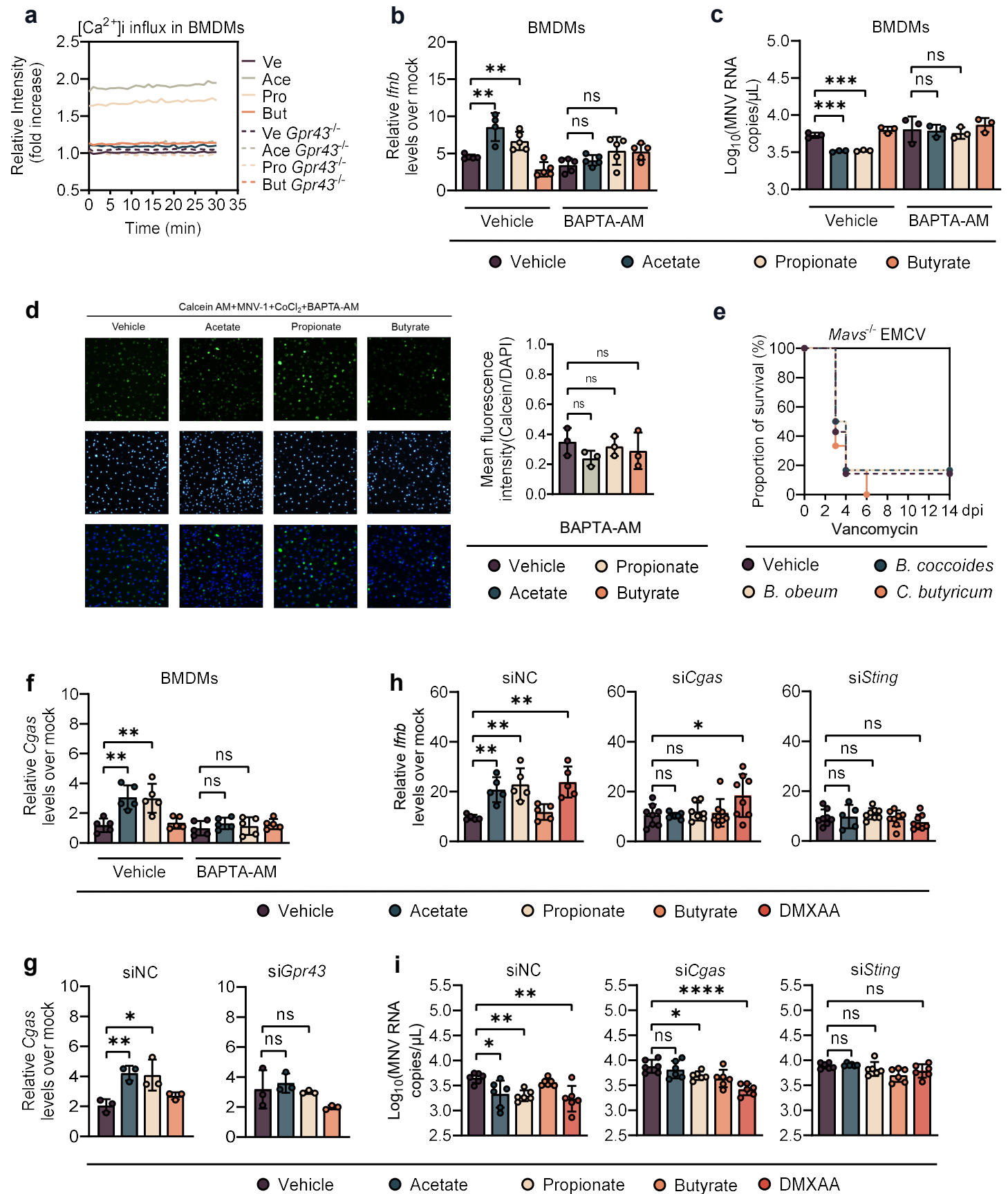

Supplement: Supplementary file 8 — extended figure8 [file 41396_2023_1541_MOESM8_ESM.pdf]
