# Supplementary material for: Ginsenoside Rg3 enriches SCFA-producing commensal bacteria to confer protection against enteric viral infection via the cGAS-STING-type I IFN axis
Source: ISME J. 2023 Nov 10;17(12):2426–40. doi: 10.1038/s41396-023-01541-7 (PMC10689736; doi:10.1038/s41396-023-01541-7)
Supplement: Supplementary file 9 — extended figure legends [file 41396_2023_1541_MOESM9_ESM.docx]

**Extended Data Fig. 1 Rg3 protects against enteric virus infection in vivo by enriching commensal bacteria. a,** Groups of mice were treated with PBS or Rg3 for 7 to 14 days before MNV infection. Du, Je, Il, Co, MLNs and feces from PBS-treated and Rg3-treated WT mice were collected at 1 dpi for viral burden analysis (n = 4). **b**, Viral burden analysis at 24 hpi in WT BMDMs pretreated for 24 h with 0, 50, 100, 150, or 200 μM Rg3 and then infected with MNV (MOI = 0.05 and 5). Cell lysates from PBS- and Rg3- treated BMDMs were collected at 24h post MNV infection for viral burden analysis (n=4 to 6). **c**, Relative abundance of bacterial families among the PBS-treated or Rg3-treated, uninfected mice. **d,** qRT-PCR analysis of bacteria genomic copies in feces from mice treated with PBS, antibiotics and Rg3 (n=5). **e**, Principal coordinates analysis among the groups of PBS, antibiotics, and Rg3 treated mice (n=5). **f**, qRT-PCR analysis of bacteria genomic copies in feces from PBS-treated or Van-treated and FMT mice (n=5 to 6). **g**, Viral burden analysis at 24 hpi in RAW264.7 cells pretreated for 24 h with 0, 50, 100, 150, or 200 μM Rg3 prior to EMCV infection (MOI =0.05 and 5) (n=4). **h,** Pathology scores at 3 dpi in Van-treated mice with or without colonization by *B. coccoides*, *B. obeum*, or *C. butyricum*  prior to i.p. infection with 2x10^2^ TCID_50_ of EMCV (n=10). **i,** Viral titers in brain and spleen tissues collected at 3 dpi from Van-treated mice with or without bacterial colonization after i.p. infection with 1x10^3^ TCID_50_ of EMCV (n=6 to 8). Plotted data represent the mean ± s.d. Unpaired two-tailed Student’s *t*-tests were used for comparison of means between treatment groups. *, *p*＜0.05; **, *p*＜0.01; ***, *p*＜0.001.

**Extended Data Fig. 2 Microbiota reconstituted in Van-treated mice or germ-free mice. a**, qRT-PCR of bacterial genomic copies in feces from PBS-treated mice or Van-treated mice with or without colonization by *B. coccoides,* *B. obeum*, or *C. butyricum* (n=6). **b**, qRT-PCR of bacterial genomic copies in feces from GF mice with or without bacterial colonization (n=5). Plotted data represent the mean ± s.d. Unpaired two-tailed Student’s *t*-tests were used for comparison of means between treatment groups. *, *p*＜0.05; ****, *p*＜0.0001.

**Extended Data Fig. 3 *Blautia* colonization in Van-treated mice upregulates SCFA levels and restricts enteric virus infection. a**, Fecal SCFA concentration of Van-treated, uninfected mice with or without colonization by *B. coccoides,* *B. obeum*, or *C. butyricum* (n=3 to 4). **b**, Correlation between fecal concentrations of acetate (left), propionate (mid), or butyrate (right) and EMCV viral burden in the spleen of Van-treated, bacteria-colonized mice (n=8). **c**, SCFA concentrations in culture supernatants of *B. coccoides,* *B. obeum*, or *C. butyricum* (n=3)*.* **d**, Fecal SCFA concentration after 2 weeks of SCFA treatment in uninfected WT mice (n=3 to 4). **e**, Pathology scores at 3 dpi in Van-treated mice pretreated with 200 mM acetate, propionate or butyrate prior to i.p. infection with 2x10^2^ TCID_50_ of EMCV (n=13 to 24). **f,** Survival curves in mice pretreated with 200 mM acetate, propionate or butyrate prior to i.p. infection with 5x10^3^ TCID_50_ of EMCV (n=10). Plotted data represent the mean ± s.d. Unpaired two-tailed Student’s *t*-tests were used for comparison of means between treatment groups. *, *p*＜0.05; **, *p*＜0.01, ****, *p*＜0.0001.

**Extended Data Fig. 4 Acetate/Propionate restrict enteric virus infection independent of adaptive immunity. a**, **b**, Flow cytometry gating strategy of (**a**) PP macrophages from MNV-infected mice at 1 dpi and **(b)** splenic macrophages from EMCV-infected mice at 3 dpi. **c**, **d**, Frequency of B-cells and T-cells from (**c**) the distal ileum PPs of MNV-infected mice at 1 dpi or (**d**) spleen of EMCV-infected mice at 3 dpi (n=5 to 6). **e**, Survival analysis of SCFA-treated WT mice given clodronate liposomes prior to i.p. inoculation with EMCV (n=5). **f**, Survival analysis of SCFA-treated Rag^-/-^ mice following i.p. inoculation with EMCV (n=5). Plotted data represent the mean ± s.d. Unpaired two-tailed Student’s *t*-tests were used for comparison of means between treatment groups.

**Extended Data Fig. 5 Acetate/propionate promote IFN-I responses in macrophages against enteric virus infection.** Viral burden analysis at 24 hpi in cell lysates from **a**, **b**, BMDMs (a) and RAW264.7 (b) cells pretreated for 24 h with 1, 5, or 10 mM acetate, propionate or butyrate prior to infection with MNV or EMCV, respectively (n=3 to 6). **c**, GO analysis comparing enriched signaling pathways at 8 hpi in MNV-infected BMDMs with or without acetate (left panel) or propionate (right panel) treatment. **d**, *Ifnb* expression in uninfected, SCFA-treated BMDMs (n=5 to 6). **e**, Relative expression of *Tnfa* (left), *Il1b* (mid) and *Il6* (right) at 8 hpi in BMDMs pretreated with SCFAs and infected with MNV (n=5 to 6). **f**, Relative expression of *Ifnb* at 8 hpi in SCFA-treated, EMCV-infected BMDMs (n=5). Plotted data represent the mean ± s.d. Unpaired two-tailed Student’s *t*-tests were used for comparison of means between treatment groups. *, *p*＜0.05; **, *p*＜0.01; ***, *p*＜0.001; ****, *p*＜0.0001; ns, not significant.

**Extended Data Fig. 6 Acetate/propionate restrict enteric virus infection by stimulating IFN-I responses in macrophages.**

**a, b,** WT and *Ifnar*^-/-^ mice were pretreated with Rg3, vancomycin (Van), or both and orally infected with MNV. **c**, **d,** WT and *Ifnar*^-/-^ mice were pretreated with Van and colonized with different bacteria prior to MNV infection. Relative *Ifnb* expression (a, c) and viral burden (b, d) were determined at 1 dpi in PP macrophages (n=5). **e**, **f**, WT and anti-IFNAR1 IgG treated mice were pretreated with Rg3, vancomycin (Van), or both and infected i.p. with EMCV. **g, h,** WT and anti-IFNAR1 IgG treated mice were pretreated with Van and colonized with different bacteria prior to i.p. EMCV infection. Survival curves (e, g) and relative *Ifnb* expression (f, h) in splenic macrophages at 3 dpi are shown (n=5). Plotted data represent the mean ± s.d. Unpaired two-tailed Student’s *t*-tests were used for comparison of means between treatment groups. *, *p*＜0.05; **, *p*＜0.01; ***, *p*＜0.001; ns, not significant.

**Extended Data Fig. 7 Acetate/propionate promotion of IFN-I is dependent on GPR43 signaling. a,** Relative expression of *Gpr41* and *Gpr43* in BMDMs from WT mice transfected with siRNA specific for *Gpr41* and *Gpr43.* (n=6). **b**, **c**, Viral titers (b) at 24 hpi and relative *Ifnb* expression (c) at 8 hpi in SCFA-pretreated BMDMs with or without knockdown of GPR41 or GPR43 expression, following MNV infection (n=6 to 8). **d**, Relative *Ifnb* expression at 8 hpi in SCFA-pretreated BMDMs collected from WT or *Gpr43*^-/-^ mice (n=5) following EMCV infection. Plotted data represent the mean ± s.d. Unpaired two-tailed Student’s *t*-tests were used for comparison of means between treatment groups. *, *p*＜0.05; **, *p*＜0.01; ***, *p*＜0.001; ****, *p*＜0.0001; ns, not significant.

**Extended Data Fig. 8 Acetate and propionate restrict enteric virus infection by activating the cGAS-STING-IFN-I axis. a**, [Ca^2+^]i influx in BMDMs isolated from WT or Gpr43^-/-^ mice was measured after 24 h incubation with acetate (Ace), propionate (Pro) or butyrate (But). Data shown are average values from three independent experiments. **b, c,** Relative *Ifnb* expression (b) at 8 hpi and viral titers (c) at 24 hpi in BMDMs treated or not with 30 µM BAPTA-AM for 24 h, incubated a further 24 h with 1 mM acetate, propionate or butyrate prior to infection with MNV (MOI = 1) (n=3 to 5). **d,** Openness of the mitochondrial permeability transition pore (mPTP) at 24 hpi was determined by mPTP kit in BMDMs pretreated with 30 µM BAPTA-AM and SCFAs followed by MNV infection. **e**, Groups of differently treated *MAVS*^-/-^ mice were i.p. infected with EMCV, and survival curves were documented (n=6 to 7). **f**, **g**, Relative *cGAS* expression at 8 hpi was analyzed in (f) control BMDMs (n=5) or (g) BMDMs transfected with siRNA specific for *Gpr43* (n=3), pretreated with BAPTA-AM and SCFAs prior to infection with MNV (MOI = 1). **h**, **i**, Relative *Ifnb* expression (h) at 8 hpi and viral titers (i) at 24 hpi were analyzed in BMDMs transfected with siRNA specific for *cGAS* or *Sting*, pretreated with SCFAs or DMXAA for 24 h prior to infection with MNV (n=6 to 8). Plotted data represent the mean ± s.d. Unpaired two-tailed Student’s *t*-tests were used for comparison of means between treatment groups. *, *p*＜0.05; **, *p*＜0.01; ***, *p*＜0.001; ****, *p*＜0.0001; ns, not significant.
